# Pathway Complexity in Supramolecular Porphyrin Self-Assembly at an Immiscible Liquid–Liquid Interface

**DOI:** 10.1021/jacs.1c02481

**Published:** 2021-06-11

**Authors:** Iván Robayo-Molina, Andrés F. Molina-Osorio, Luke Guinane, Syed A. M. Tofail, Micheál D. Scanlon

**Affiliations:** † The Bernal Institute and Department of Chemical Sciences, School of Natural Sciences, University of Limerick (UL), Limerick V94 T9PX, Ireland; ‡ The Bernal Institute and Department of Physics, School of Natural Sciences, University of Limerick (UL), Limerick V94 T9PX, Ireland; § Advanced Materials and Bioengineering (AMBER) Centre, CRANN Institute, Trinity College Dublin (TCD), Dublin 2 D02 PN40, Ireland

## Abstract

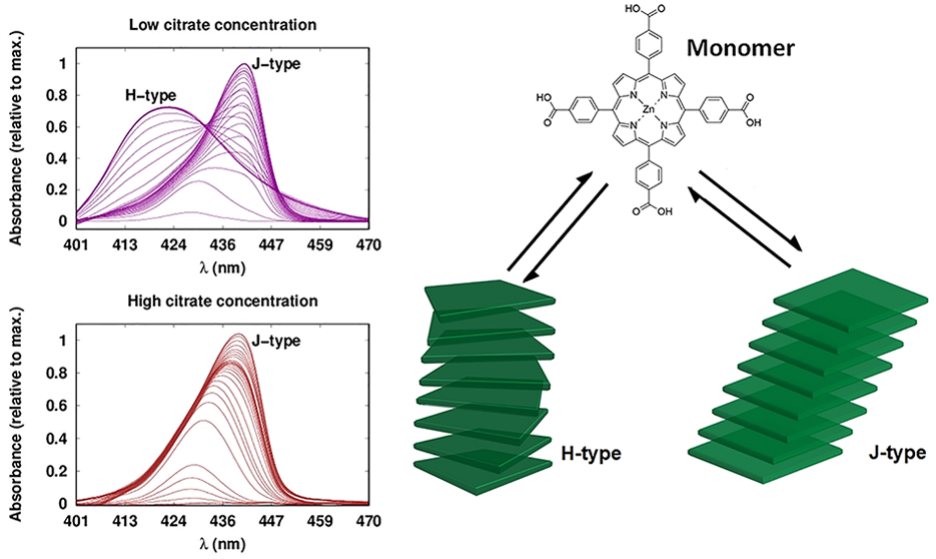

Nanostructures that are inaccessible through spontaneous thermodynamic processes may be formed by supramolecular self-assembly under kinetic control. In the past decade, the dynamics of pathway complexity in self-assembly have been elucidated through kinetic models based on aggregate growth by sequential monomer association and dissociation. Immiscible liquid–liquid interfaces are an attractive platform to develop well-ordered self-assembled nanostructures, unattainable in bulk solution, due to the templating interaction of the interface with adsorbed molecules. Here, we report time-resolved *in situ* UV–vis spectroscopic observations of the self-assembly of zinc(II) meso-tetrakis(4-carboxyphenyl)porphyrin (ZnTPPc) at an immiscible aqueous–organic interface. We show that the kinetically favored metastable J-type nanostructures form quickly, but then transform into stable thermodynamically favored H-type nanostructures. Numerical modeling revealed two parallel and competing cooperative pathways leading to the different porphyrin nanostructures. These insights demonstrate that pathway complexity is not unique to self-assembly processes in bulk solution and is equally valid for interfacial self-assembly. Subsequently, the interfacial electrostatic environment was tuned using a kosmotropic anion (citrate) in order to influence the pathway selection. At high concentrations, interfacial nanostructure formation was forced completely down the kinetically favored pathway, and only J-type nanostructures were obtained. Furthermore, we found by atomic force microscopy and scanning electron microscopy that the J- and H-type nanostructures obtained at low and high citric acid concentrations, respectively, are morphologically distinct, which illustrates the pathway-dependent material properties.

## Introduction

Self-assembly is a powerful route to access elaborate, functional supramolecular nanostructures from relatively simple molecules.^1−4^ The properties of these nanostructures, and ensuing performance characteristics in device applications, depend on the precise molecular organization of the individual building blocks.^5^ Supramolecular polymers are a key subclass of self-assembled nanostructures, defined as one-dimensional arrays of monomeric units that are interconnected by reversible and highly directional secondary interactions such as hydrogen bonds, metal–ligand coordination, π–π stacking, or combinations thereof.^6,7^

Over the past decade, kinetic studies probing the time-dependent behavior of supramolecular polymers composed of, for example, porphyrin,^8−12^ bis(merocyanine),^13^ oligo(*para*-phenylenevinylene),^14,15^ or perylene bisimide dyes,^16−18^ have comprehensively demonstrated the existence of competing assembly pathways, that is, pathway complexity. Control over the interplay between these competing pathways is heavily influenced by the preparation methodologies (concentration, temperature, pH, solvent, ionic strength, external stimuli, etc.).^6,19^ Thus, manipulation of the latter can potentially lead to nanostructures formed at the thermodynamic equilibrium of the system, or alternatively metastable or kinetically trapped nonequilibrium nanostructures.^6,19^

The competing assembly pathways that lead to supramolecular polymers can be described by distinct isodesmic or cooperative (nucleation–elongation) mechanisms.^19−23^ In an isodesmic mechanism, the Gibbs free energy of every monomer addition is equivalent, with all individual steps described by a single equilibrium constant (*K*).^24^ A cooperative mechanism is characterized by formation of a thermodynamically unfavorable nucleus (or oligomer), followed by energetically favored elongations steps, and described by two equilibrium constants for the nucleation (*K*_n_) and elongation steps (*K*_e_), respectively.^21^ These mechanisms have been distinguished by concentration- and/or temperature-dependent spectroscopic measurements that probe the molecule to nanostructure transition.^23^

To date, pathway complexity has been described exclusively for systems that self-assemble in bulk solutions, although there has also been some interesting work conducted at solid–liquid interfaces. For instance, these interfaces have been used as a template for polymer growth^25^ or as a platform to measure polymer growth using high-speed atomic force microscopy (AFM).^26−28^ A powerful alternative approach is molecular self-assembly at “soft” liquid–air or immiscible liquid–liquid interfaces.^29−32^ Such “soft” interfaces are considered defect-free, highly reproducible, and self-healing.^33^ These attributes facilitate macroscale uniformity in molecule–interface interactions, providing a route to self-assembled films of nanomaterials with continuous domains of macroscale (>cm^2^) long-range order, exhibiting high structural perfection.^34^

Due to their similarities to natural dyes functioning in photosynthetic systems, supramolecular assemblies of zinc(II) 5,10,15,20-(tetra-4-carboxyphenyl)porphyrin (ZnTPPc) molecules at immiscible liquid–liquid interfaces are of particular interest for solar energy conversion and storage applications.^35,36^ Early work demonstrated that photocurrents obtained at porphyrin nanostructure functionalized liquid–liquid interfaces are remarkably dependent on the light polarization, indicating a well-ordered self-assembled nanostructure due to the templating interaction of the interface.^37^ Recently, our group demonstrated that these interfacial ZnTPPc nanostructures are stabilized by cooperative hydrogen bonding and likely represent metastable or kinetically trapped nonequilibrium nanostructures.^38,39^

Despite such insights, our understanding of the assembly mechanism of nanostructures at immiscible liquid–liquid interfaces remains limited. Due to easily detectable spectral changes arising from exciton coupling of their transition dipole moments, dye molecules are ideal candidates to study the mechanisms and thermodynamics of interfacial self-assembly processes by UV–vis spectroscopy. Here we report time-resolved UV–vis spectroscopic observations of the formation of supramolecular assemblies of ZnTPPc at an immiscible liquid–liquid interface as a function of the aqueous pH, porphyrin concentration, and electrolyte concentration. Due to the interface’s buried nature, we developed a custom UV–vis setup that operates in total internal reflection mode (TIR-UV–vis absorption) to monitor *in situ* the evolution with time of the Soret band of adsorbed interfacial ZnTPPc species. Multiple ZnTPPc nanostructures formed on the interface simultaneously lead to overlapping of their spectrophotometric signals. Thus, the spectral data were analyzed by a multivariate curve resolution with alternating least squares (MCR-ALS) decomposition methodology. Quantitative insight into the kinetic experiments was obtained from kinetic model calculations (isodesmic and cooperative, respectively), which revealed two parallel and competing pathways leading to the different ZnTPPc nanostructures. Finally, the citric acid concentration in the aqueous phase was increased to change the chemical environment of the self-assembly process and influence the pathway selection.

## Results and Discussion

### Spectroscopically Monitoring the pH- and Concentration Dependency of ZnTPPc Interfacial Self-Assembly

As discussed in our previous work,^39^ ZnTPPc self-assembles at the interface between water and an immiscible organic solvent, such as α,α,α-trifluorotluene (TFT), to form highly ordered nanostructures. The self-assembly process is selective to the interface, only taking place when the aqueous pH is within ±0.2 units of the p*K*_a_ of the porphyrin’s carboxyl groups (pH 5.8).^40^ The electronic transitions of the porphyrin’s Soret band, observed between 410 and 470 nm, are sensitive to its molecular environment and, thus, aggregation state. Therefore, by monitoring the Soret band absorbance *in situ* at the interface with time (up to 1000 s) by TIR-UV–vis absorption (see experimental setup in Figure S1), we probed the influence of the preparation methodology in terms of pH, bulk aqueous ZnTPPc concentration ([ZnTPPc]_aq_), and aqueous electrolyte concentration on the interfacial self-assembly kinetics of ZnTPPc.

**Figure 1:**
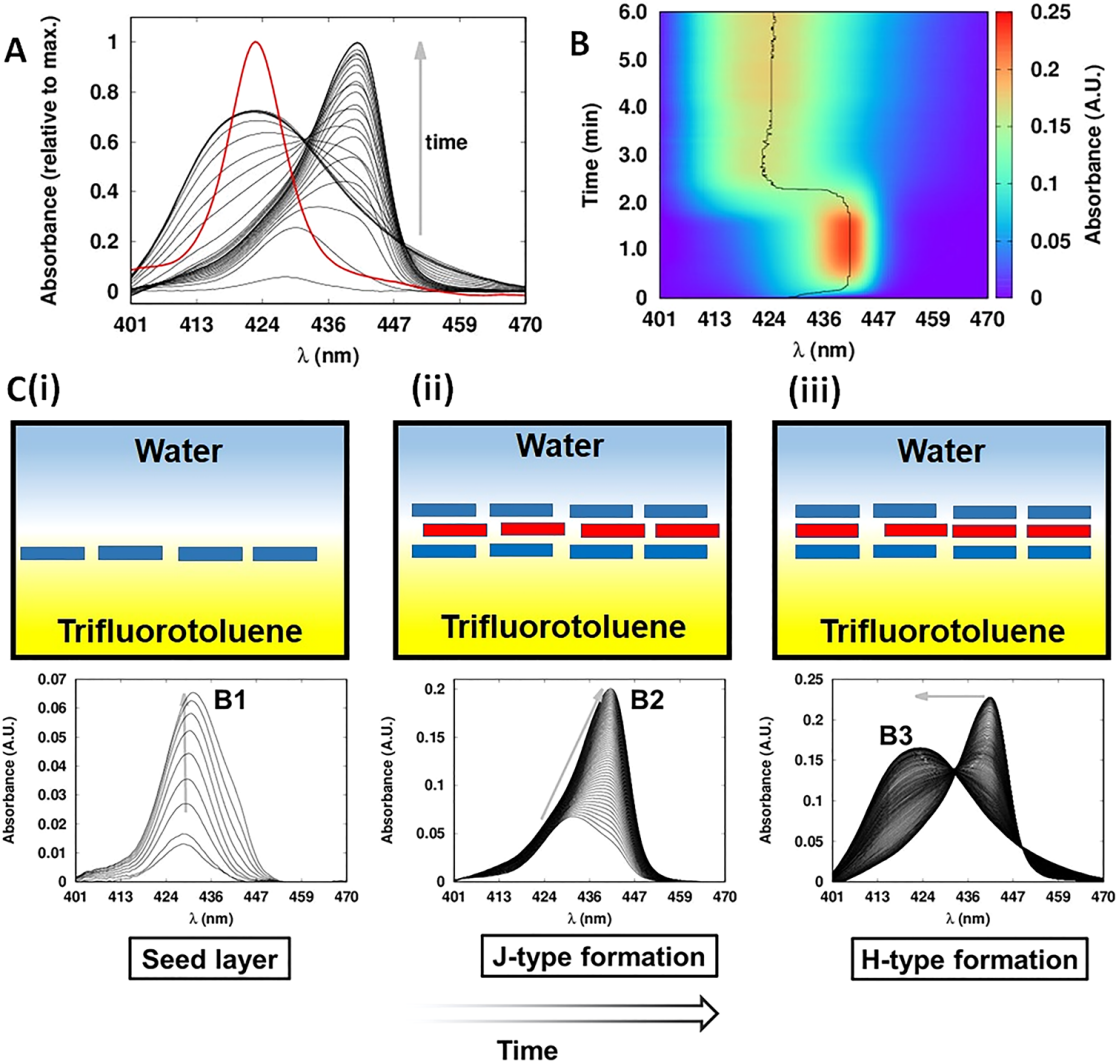
Time-dependent TIR-UV–vis spectra of ZnTPPc interfacial self-assembly at an immiscible aqueous–organic interface. (A) The bulk aqueous ZnTPPc concentration ([ZnTPPc]_aq_) was 8 μM, the aqueous electrolyte employed was 10 mM citric acid, and the pH was adjusted to 5.8. The organic phase was neat TFT. TIR-UV–vis spectra were taken every 0.5 s for 500 s (every 10th spectra is shown for clarity). The red spectrum is that of bulk aqueous ZnTPPc at pH 5.8. The raw spectra were treated in R^42^ using the package baseline^43^ for smoothing and correcting the drift of the signal (Figure S2). (B) Heat-map of the absorbance between 400 and 470 nm with time, clearly showing the trends in the shift of the λ_max_ as the dominant ZnTPPc species at the interface changes with time. (C) Schematic representation of the self-assembling behavior of ZnTPPc at the aqueous–organic interface. The three-stages of self-assembly were identified as (i) adsorption of monomeric ZnTPPc at the aqueous–organic interface to form a “seed layer” (designated Soret 1, or B1, with a λ_max_ of 430 nm), (ii) rapid formation of metastable J-type nanostructures (B2, λ_max_ of 442 nm), and (iii) partial interconversion of the J-type to a H-type nanostructure (B3, λ_max_ of 418 nm). The associated TIR-UV–vis spectra from (A) are shown below each schematic, and arrows indicate the general shift in the λ_max_ as the dominant spectral features (B1, B2, or B3) evolve with time. An animated version is displayed in the Supporting Information (SI).

The absorbance spectra at pH 5.8 with 8 μM ZnTPPc added to the bulk aqueous phase evolved with time, strongly indicating the formation of multiple interfacial nanostructures (Figure 1A). These spectra can be divided into three sequential steps, each clearly identifiable on the TIR-UV–vis spectra heat-map in Figure 1B. First, a growing band (denoted as B1) with a λ_max_ at 430 nm was observed (Figure 1C(i)). Given the presence of this band at other pH conditions (discussed *vide infra*) and the λ_max_ of ZnTPPc molecules in solution (422 nm at pH 5.8), we attributed B1 to individual ZnTPPc molecules adsorbed at the aqueous–organic interface. These adsorbed molecules can serve as a seed layer for further nanostructure growth. Second, another growing band (B2) with a λ_max_ at 442 nm was observed (Figure 1C(ii)). Being red-shifted from B1, this band was associated with the formation of an initial J-type interfacial nanostructure. Finally, a third growing band (B3) with a λ_max_ at 418 nm appeared (Figure 1C(iii)) and was attributed to the formation of a H-type interfacial nanostructure. These final spectra were quite broad, indicating signal overlapping from multiple interfacial nanostructures. Furthermore, the formation of B3 implied the presence of an isosbestic point at 433 nm and, thus, that partial H–J structural interconversion did not require an intermediate species.^41^

Of the parameters evaluated, the self-assembly process was most sensitive to the aqueous pH, in agreement with our previous findings.^39^ A range of pH values were investigated between pH 5.0 and 6.8 with 8 μM ZnTPPc added to the bulk aqueous phase (Figure S3). For control experiments in the absence of ZnTPPc, no UV–vis signal was detected in the region of interest. Upon addition of ZnTPPc at pH values marginally (≥0.3 pH units) more acidic or alkali than the p*K*_a_, a single band with a λ_max_ at 430 nm was observed (Figure S3). These bands at 430 nm were distinct from those associated with the bulk aqueous ZnTPPc molecules at each pH value, shown as red spectra in Figure S3, and instead attributed to ZnTPPc monomers adsorbed at the aqueous–organic interface. A strikingly different behavior was observed at pH 5.8 (Figure 1A), with an evolution of the absorbance spectra with time that strongly indicated the formation of multiple interfacial nanostructures.

To study the effect of porphyrin concentration on the self-assembly kinetics, TIR-UV–vis spectra were analyzed by varying the bulk aqueous ZnTPPc concentration ([ZnTPPc]_aq_) between 1 and 10 μM at optimal pH 5.8 conditions (Figure S4). Using the isotherm of this biphasic system at pH 5.8,^39^ these [ZnTPPc]_aq_ values led to interfacial ZnTPPc concentrations (Γ_[ZnTPPc]_)) between 0.4 and 4.8 nmol·cm^–2^, respectively. At Γ_[ZnTPPc]_ < 2.6 nmol·cm^–2^, only the Soret band of adsorbed ZnTPPc was detected with no change in band intensity after 600 s (Figure S4A,B). Meanwhile, at Γ_[ZnTPPc]_ > 5 nmol·cm^–2^, the volume of the aliquot injected into the system destabilized the baseline and thus inhibited the acquisition of UV–vis spectra under TIR conditions. Therefore, to ensure statistically robust TIR-UV–vis spectra acquisition, the Γ_[ZnTPPc]_ range was limited between 0.4 and 5 nmol·cm^–2^ (in effect a [ZnTPPc]_aq_ range between 5 and 10 μM). Within this selected concentration range, interfacial self-assembly proceeded through the three-stage mechanism discussed *vide supra*.

### Kinetic Modeling of Interfacial ZnTPPc Self-Assembly by MCR-ALS Analysis

**Figure 2:**
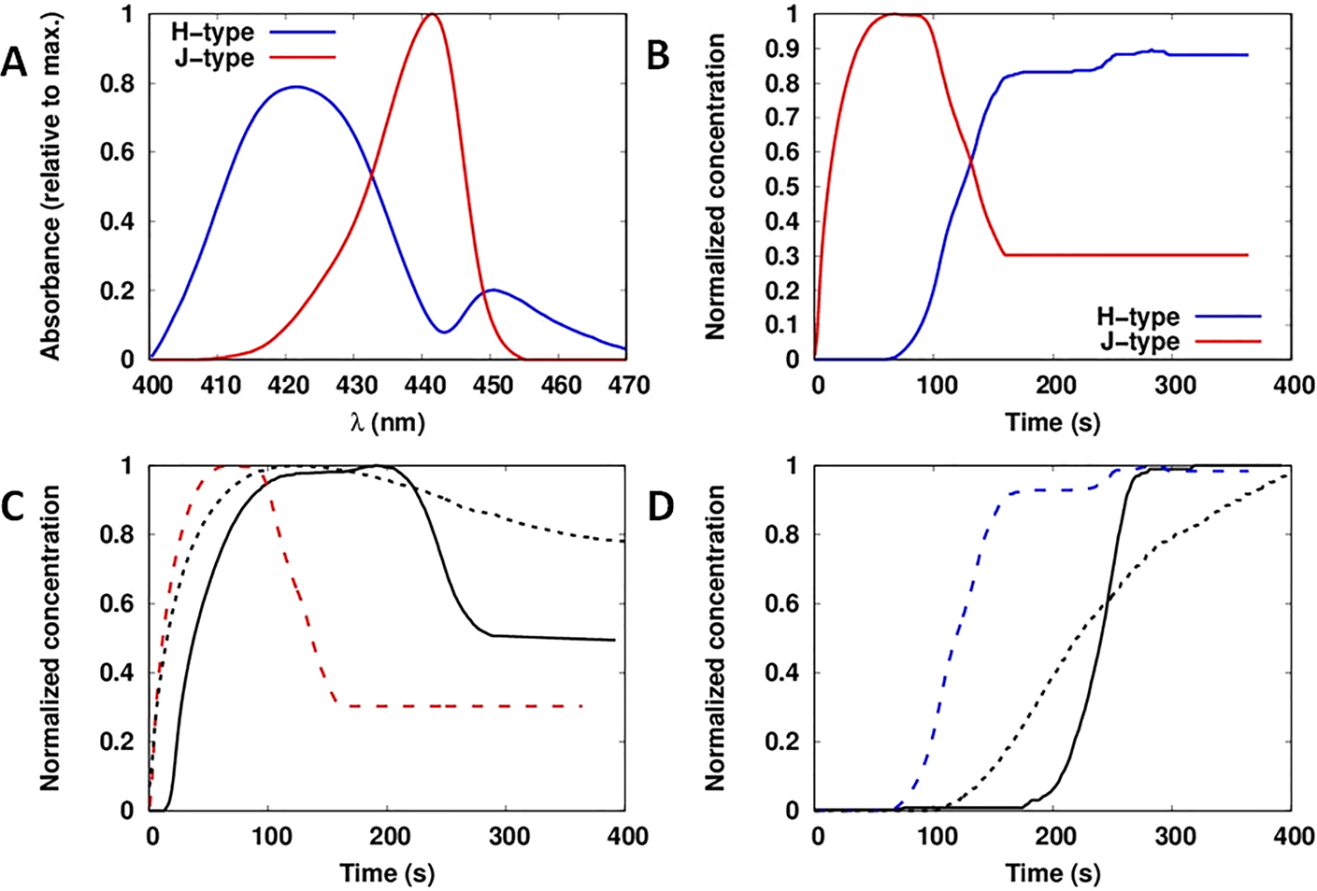
MCR-ALS analysis of the kinetics of interfacial ZnTPPc self-assembly. (A) MCR-ALS resolved the pure spectra of the H- and J-type nanostructures for an interfacial ZnTPPc concentration (Γ_[ZnTPPc]_) value of 4 nmol·cm^–2^_._ at pH 5.8 and (B) the corresponding kinetic profiles for the H- and J-type nanostructures, respectively. (C, D) Comparison of the kinetic profiles resolved by MCR-ALS for Γ_[ZnTPPc]_ values of 2.6 (solid line), 4.0 (dashed line), and 4.8 nmol·cm^–2^ (dotted line), respectively, for (C) the J-type nanostructure and (D) the H-type nanostructure. The quality control parameters of the MCR-ALS modeling are detailed in Table S1.

Due to a severe overlapping of the spectrophotometric signals (from B1, B2 and B3 discussed above), a principal component analysis (PCA) was first applied to the TIR-UV–vis spectra obtained at pH 5.8 for Γ_[ZnTPPc]_ values of 2.6, 4.0, and 4.8 nmol·cm^–2^, respectively. The scree plot and representative PCA results for 4.0 nmol·cm^–2^ are shown in Figure S5. The analysis revealed two significant interfacial ZnTPPc species, identified as H- and J-type nanostructures, with λ_max_ of 418 and 442 nm, respectively. The abstract spectra extracted by PCA of each species for a Γ_[ZnTPPc]_ of 4.0 nmol·cm^–2^ were used as a starting point (Figure S5C). An MCR-ALS analysis was run to resolve the pure spectra and kinetic profile of each species. The resulting concentration profiles show that the interfacial J-type nanostructures rapidly formed, reaching a maximum concentration after 50 s (Figure 2B). The H-type nanostructures formed slower and presented a clear lag-time, suggesting their formation through a nucleated growth mechanism.^23^ In addition, the growth of H-type nanostructures was accompanied by a decrease in the concentration of J-type until their concentrations equilibrated after 250 s (Figure 2B). The corresponding pure spectra extracted by MCR-ALS analysis for Γ_[ZnTPPc]_ values of 2.6 and 4.8 nmol·cm^–2^ are shown in Figure S6. The quality control parameters of the MCR-ALS modeling are detailed in Table S1.

Comparisons of the influence of [ZnTPPc]_aq_ on the behavior of the kinetic profiles for the J- and H-type nanostructures, respectively, are shown in Figure 2C,D. The J-type nanostructure presented a small lag-time for formation only at the lower Γ_[ZnTPPc]_ of 2.6 nmol·cm^–2^ (Figure 2C). Increasing Γ_[ZnTPPc]_ from 2.6 to 4.0 nmol·cm^–2^ significantly decreased the lag-time for H-type formation (Figure 2D). The kinetic profiles for the higher Γ_[ZnTPPc]_ of 4.8 nmol·cm^–2^ were qualitatively similar but out of sequence with the 2.6 and 4 nmol·cm^–2^ profiles. This was attributed to the greater difficulty in isolating the pure spectra by PCA analysis due to the rapid enhancement in the overlapping of the spectrophotometric signals of the individual interfacial nanostructures as Γ_[ZnTPPc]_ increased.

### ODE-Based Kinetic Modeling

The kinetic profiles in Figure 2C,D evidence the existence of two distinct interfacial ZnTPPc nanostructures, but the details of the interconversion mechanism between the J- and H-type species were not immediately evident. Based on the MCR-ALS analysis, two mechanisms can be proposed: (i) direct conversion from J- to H-type nanostructures or (ii) via two parallel pathways where both nanostructures compete for free monomers. Although the direct conversion mechanism is intuitively attractive, recent reports have demonstrated that competitive pathways in supramolecular polymerization are an increasingly observed phenomenon.^6,12,19,44−46^

**Figure 3:**
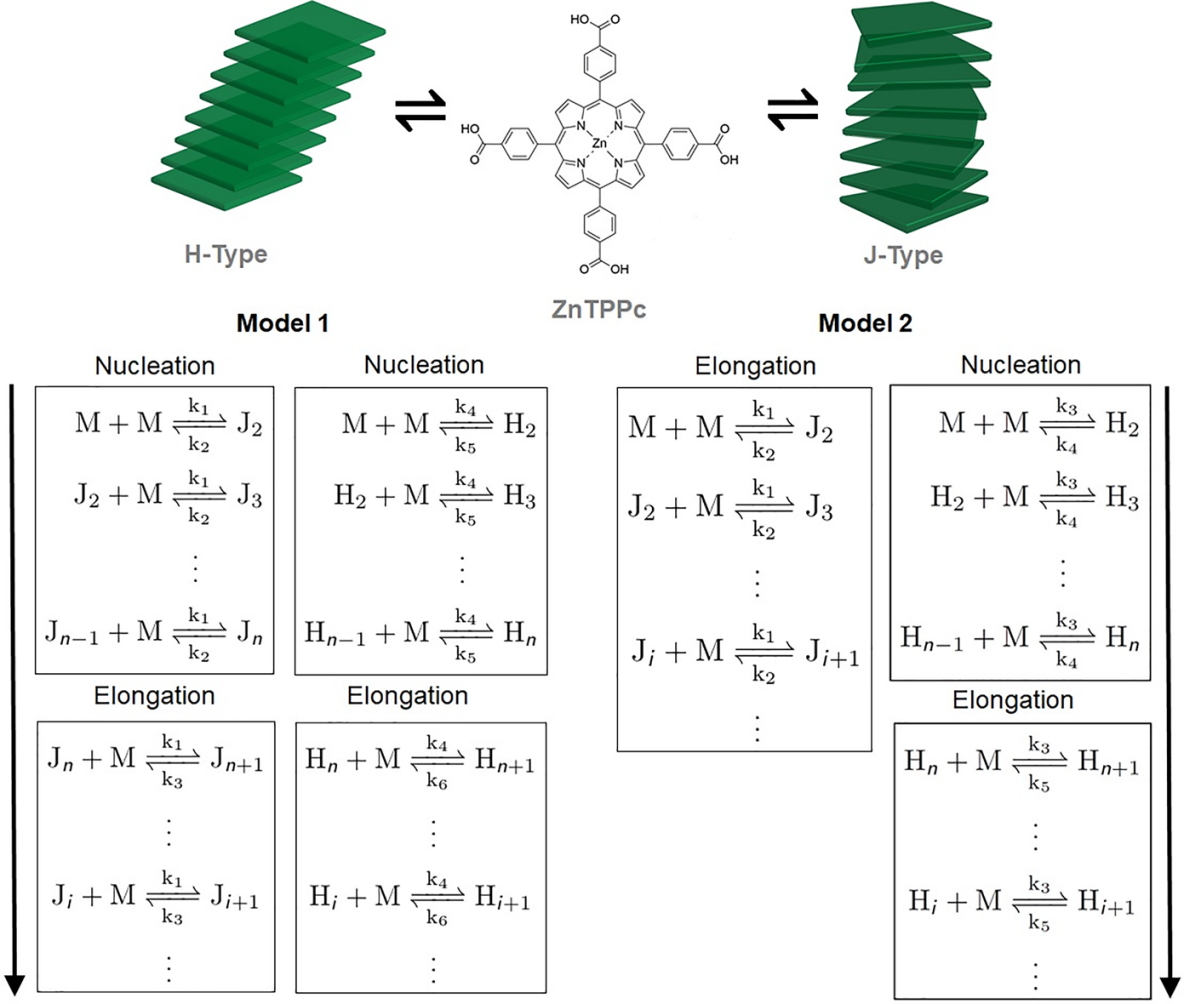
Kinetic models explored to simulate porphyrin supramolecular polymerization (or self-assembly) through two coupled pathways competing for the porphyrin monomers at the immiscible liquid–liquid interface. The kinetic models are based on monomer association and dissociation of a supramolecular polymerization consisting of two coupled cooperative (nucleation-elongation) pathways (model 1) or an isodesmic pathway coupled with a cooperative pathway (model 2). Models 1 and 2 employ a total of 6 and 5 rate constants, respectively, as explained in detail in the SI.

Two kinetic models based on ordinary differential equations (ODE) were developed. These models are summarized in Figure 3. In model 1, two competitive cooperative (nucleation–elongation) pathways were coupled, whereas in model 2, an isodesmic pathway competed with a cooperative pathway. Model 1 employs 6 rate constants, while model 2 employs 5 rate constants. Model 1 indicates that, regardless of Γ_[ZnTPPc]_, the J-type nanostructure should present a small induction period as evident for the kinetic profile of the most dilute Γ_[ZnTPPc]_ value of 2.6 nmol·cm^–2^ (Figure 2C). In general, these kinetic models describe the rate of change of the interfacial nanostructure (or aggregate) concentration using the following ODE:
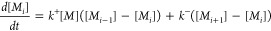
1where [*M*_*i*_] is the concentration of a nanostructure of length *i*, and *k*^+^ and *k*^–^ are the association and dissociation rate constants, respectively. The first term of the equation accounts for the nanostructure growing by monomer association, while the second term accounts for the nanostructure shrinking by monomer dissociation. A detailed description of the kinetic modeling procedure and an overview of the full ODE systems specifying the exact reaction steps involved are provided in the SI.

**Figure 4:**
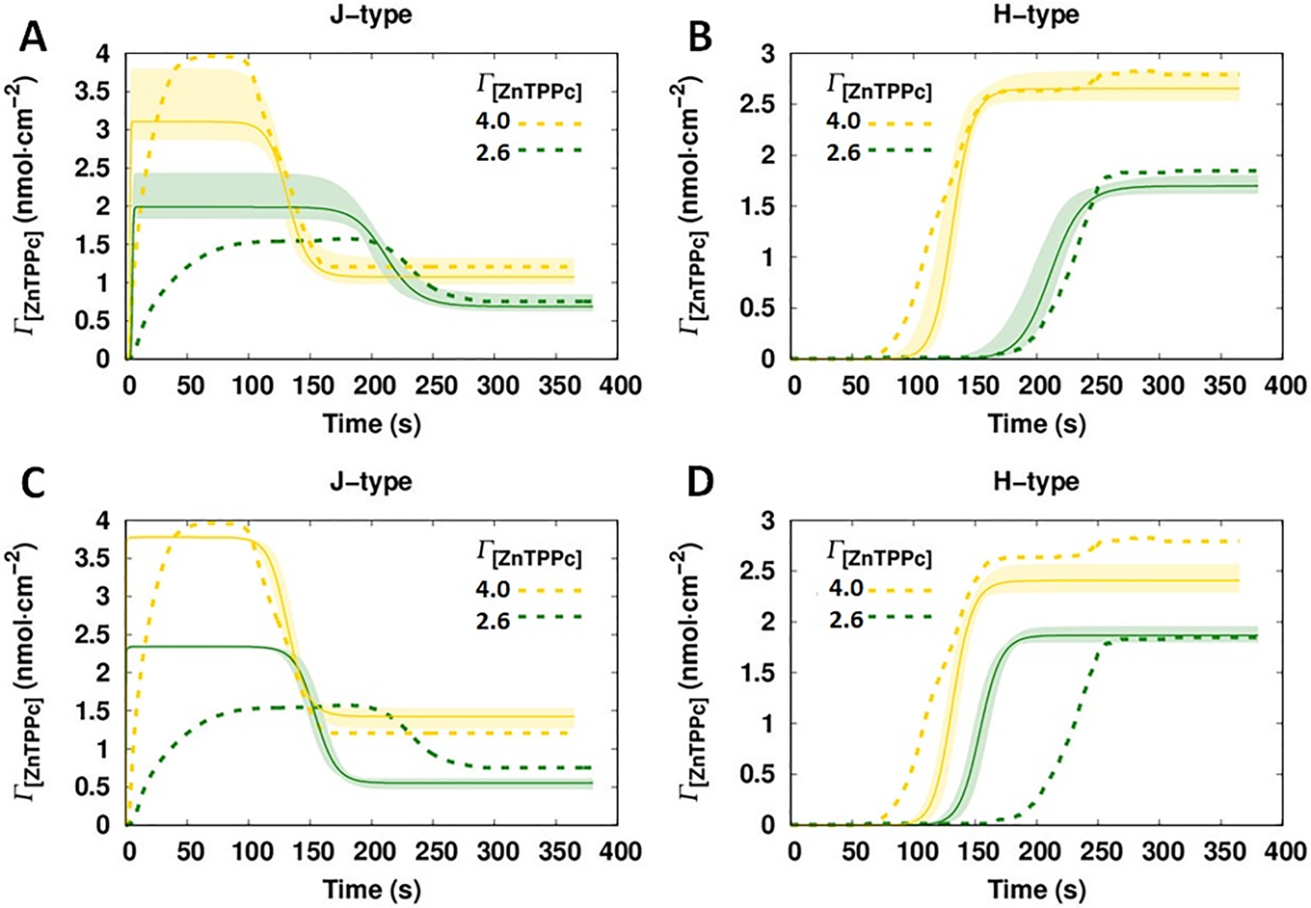
Extracting the kinetic constants from the MCR-ALS analysis of interfacial ZnTPPc self-assembly. Two models were explored (see Figure 3 and the SI), and for both models, the kinetic constants were extracted from the best-fits obtained by a global fitting using the kinetics profiles resolved at Γ_[ZnTPPc]_ values of 2.6 and 4.0 nmol·cm^–2^. Time–concentration curves from (A, B) model 1 and (C, D) model 2 for the J- and H-type nanostructures (solid lines) were compared with the kinetic profiles obtained by MCR-ALS analysis (dashed lines). The shadowed areas indicate the sensitivity range based on the parameter distributions generated using the MCMC method. Parameter values determined by MCMC for models 1 and 2 are presented in Table 1. Further information regarding the parameter distribution can be found in the SI (Tables S2–S6 and Figures S7–S12).

Kinetic constants for both models were extracted from the Γ_[ZnTPPc]_ profiles for the data set obtained at pH 5.8 using a global fitting of the Γ_[ZnTPPc]_ values of 2.6 and 4 nmol·cm^–2^, see Figure 4. At these conditions, the interfacial concentrations of both the J- and H-type nanostructures reached a stable equilibrium after 150 s. The Γ_[ZnTPPc]_ value of 5 nmol·cm^–2^ was omitted because the kinetic profile of the H-type nanostructure does not present a well-defined sigmoidal shape (Figure 2B). The Markov chain Monte Carlo (MCMC) method was selected for the fitting procedure given its robust predictions based on the parameters uncertainty.^47^ This aspect is of paramount importance, as the main issue affecting the resolution of bilinear data in MCR is the nonunicity of the solution due to rotational and intensity ambiguities of the solution.^48−50^ The use of constraints can diminish these ambiguities, although it does not eliminate them completely. Solutions in MCR are usually represented as feasible bands.^51^

To scale the solutions obtained by MCR-ALS, it was assumed that at equilibrium, all porphyrin monomers were either as an H- or J-type nanostructure, and therefore the mass balances for models 1 and 2 were defined as follows:
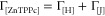
2
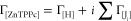
3Eqs 2 and 3 correspond to models 1 and 2, respectively. In model 2, as the J-type nanostructure is formed through an isodesmic model, the total interfacial concentration is given by the term *i*∑Γ_[J_*i*_]_. The fitting process using MCMC is described in detail in the SI.

Parameter values determined by MCMC for models 1 and 2 are presented in Table 1. Further information regarding the parameter distribution can be found in the SI (Tables S2–S6 and Figures S7–S12). For model 1, the values clearly show that the nucleation constant (*K*_n_ = *k*_1_/*k*_2_ = 3.71 × 10^–6^ cm^2^·nmol^–1^) of the J-type nanostructure is 2 orders of magnitude larger than the nucleation constant of the H-type nanostructure (*K*_n_ = *k*_4_/*k*_5_ = 3.10 × 10^–8^ cm^2^·nmol^–1^). In contrast, the elongation constant of the J-type nanostructure (*K*_e_ = *k*_1_/*k*_3_ = 3.06 cm^2^·nmol^–1^) is 2.5 times smaller than the elongation constant of the H-type nanostructure (*K*_e_ = *k*_4_/*k*_6_ = 7.80 cm^2^·nmol^–1^). For model 2, *K*_n_ of the H-type nanostructure is 6.75 × 10^–3^ cm^2^·nmol^–1^, while *K*_e_ of the H-type nanostructure is 4.3 times bigger than *K*_e_ of the J-type nanostructure (7.05 and 1.65 cm^2^·nmol^–1^ for the J- and H-type, respectively).

**Table 1:** Optimized Parameter Values Obtained by the MCMC Algorithm Using Model 1 (Two Coupled Cooperative Pathways) and Model 2 (Coupled Isodesmic and Cooperative Pathways), Respectively^a^

model 1						
	cooperative (nucleation–elongation) pathway			cooperative (nucleation–elongation) pathway		
	*k*_1_ (cm^2^·nmol^–1^·s^–1^)	*k*_2_ (s^–1^)	*k*_3_ (s^–1^)	*k*_4_ (cm^2^·nmol^–1^·s^–1^)	*k*_5_ (s^–1^)	*k*_6_ (s^–1^)
value	9.83 × 10^–1^	2.65 × 10^5^	3.21 × 10^–1^	1.29 × 10^–1^	4.17 × 10^7^	1.66 × 10^–2^
SD^b^	7.80 × 10^–3^	9.49 × 10^4^	8.84 × 10^–2^	8.56 × 10^–2^	9.75 × 10^7^	2.05 × 10^–3^

model 2						
	isodesmic pathway			cooperative (nucleation–elongation) pathway		
	*k*_1_ (cm^2^·nmol^–1^·s^–1^)	*k*_2_ (s^–1^)		*k*_3_ (cm^2^·nmol^–1^·s^–1^)	*k*_4_ (s^–1^)	*k*_5_ (s^–1^)
value	1.65	1.00		2.25 × 10^–1^	3.34 × 10^1^	3.19 × 10^–2^
SD^b^	9.30 × 10^–2^	1.40 × 10^–3^		4.42 × 10^–2^	1.72 × 10^3^	5.42 × 10^–3^

a Further information regarding the parameter distribution can be found in the SI (Tables S2–S6 and Figures S7–S12).

b Standard deviation.

To further investigate the parameter uncertainty found by MCMC, a sensitivity analysis was completed (see Tables S5 and S6). The sensitivity coefficients as a function of time for both interfacial ZnTPPc nanostructures are shown in Figures S11 and S12. Both models present similar results; in the case of the J-type nanostructure, the association constant for this aggregate (*k*_1_ for both models) has a positive effect over the formation of this nanostructure. Meanwhile, the association of the H-type and dissociation of the J-type (*k*_4_ and *k*_2_, for model 1 and *k*_3_ and *k*_2_ for model 2, respectively) have a negative effect. In contrast, H-type nanostructures present the opposite trend. These results clearly show how these pathways are competing. Additionally, Figures S11 and S12 show that the output for both models is more sensitive to the parameters when the H-type nanostructure starts to rise sharply. Finally, it is clearly seen that the sensitivity of the nucleation constant for H-type in model 1 (*k*_5_) is small for both nanostructures. Hence, the value of this parameter can change considerably, and the effect to the output is small.

The best-fittings found by MCMC, and overlaid by the MCR-ALS result (dashed line) for each nanostructure, are shown for model 1 in Figure 4A,B and model 2 in Figure 4C,D. Clearly, model 1 can reproduce the formation of the H-type nanostructure accurately (Figures 4B). However, for the J-type nanostructure, only the last part of the process is described reasonably (Figure 4A). On the other hand, model 2 only can only describe the curves obtained with a Γ_[ZnTPPc]_ value of 4.0 nmol·cm^–2^, and, in contrast, with a Γ_[ZnTPPc]_ value of 2.6 nmol·cm^–2^, the match is poorly described by model 2.

**Figure 5:**
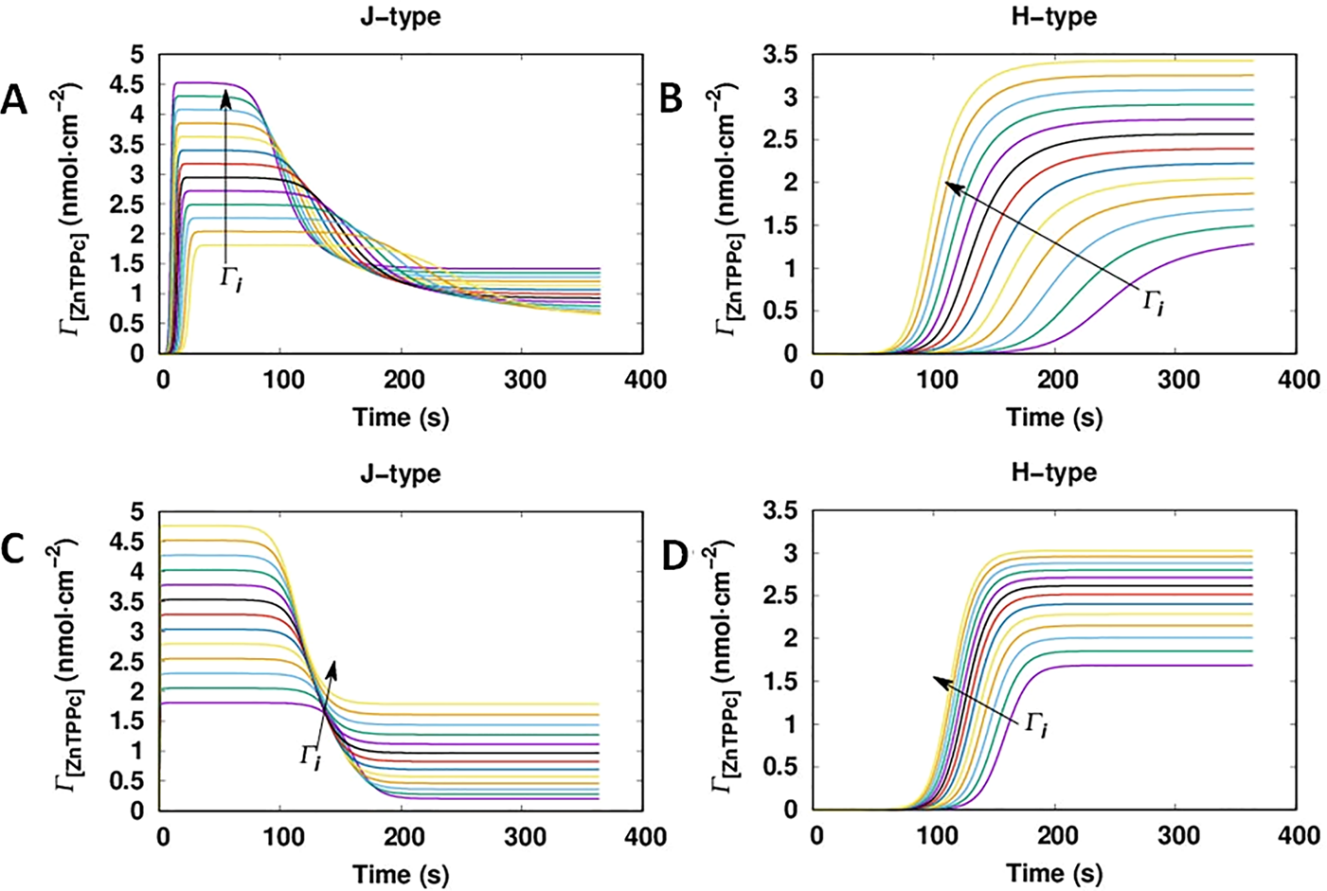
Simulation of the Γ_[ZnTPPc]_ kinetic profiles as a function of increasing Γ_[ZnTPPc]_. The time–concentration curves from (A, B) model 1 and (C, D) model 2 for the J- and H-type nanostructures, respectively, were simulated using the parameter values determined by MCMC for each model (shown in Table 1). Γ_[ZnTPPc]_ was varied from 2 to 5 nmol·cm^–2^. The arrow indicates the direction of increasing interfacial concentration.

To determine the dependence of the kinetic profiles on Γ_[ZnTPPc]_, time–concentration curves for the J- and H-type nanostructures for both models were simulated using the values shown in Table 1 (Figure 5). Model 1 predicted a strong interfacial concentration dependence of the lag-time for the formation of the H-type nanostructure (Figure 5B). This period is reduced by more than 100 s when Γ_[ZnTPPc]_ increased from 2 to 5 nmol·cm^–2^, a range covered by our experimental data in Figure 2. In the case of the J-type nanostructure, the induction period slightly increased with the concentration. In contrast, for model 2, the dependence of the kinetic profiles on Γ_[ZnTPPc]_ was weak. The induction period of the H-type nanostructure changed by <50 s when Γ_[ZnTPPc]_ increased from 2 to 5 nmol·cm^–2^. In the same way, the kinetic profile for the J-type nanostructure was relatively unaffected. Thus, the experimentally observed dynamic behavior found by MCR-ALS in Figure 2 was better described by model 1: two cooperative pathways competing for the free monomers adsorbed at the liquid–liquid interface. It is worth nothing that while the current two-pathway model provides a minimal description of the experimental observations, the actual system may involve additional equilibria such as fragmentation and coagulation^52^ or the diffusion of ZnTPPc across the interface.

### Modifying Pathway Selection to Favor the Formation of the Metastable J-Type Nanostructure

**Figure 6:**
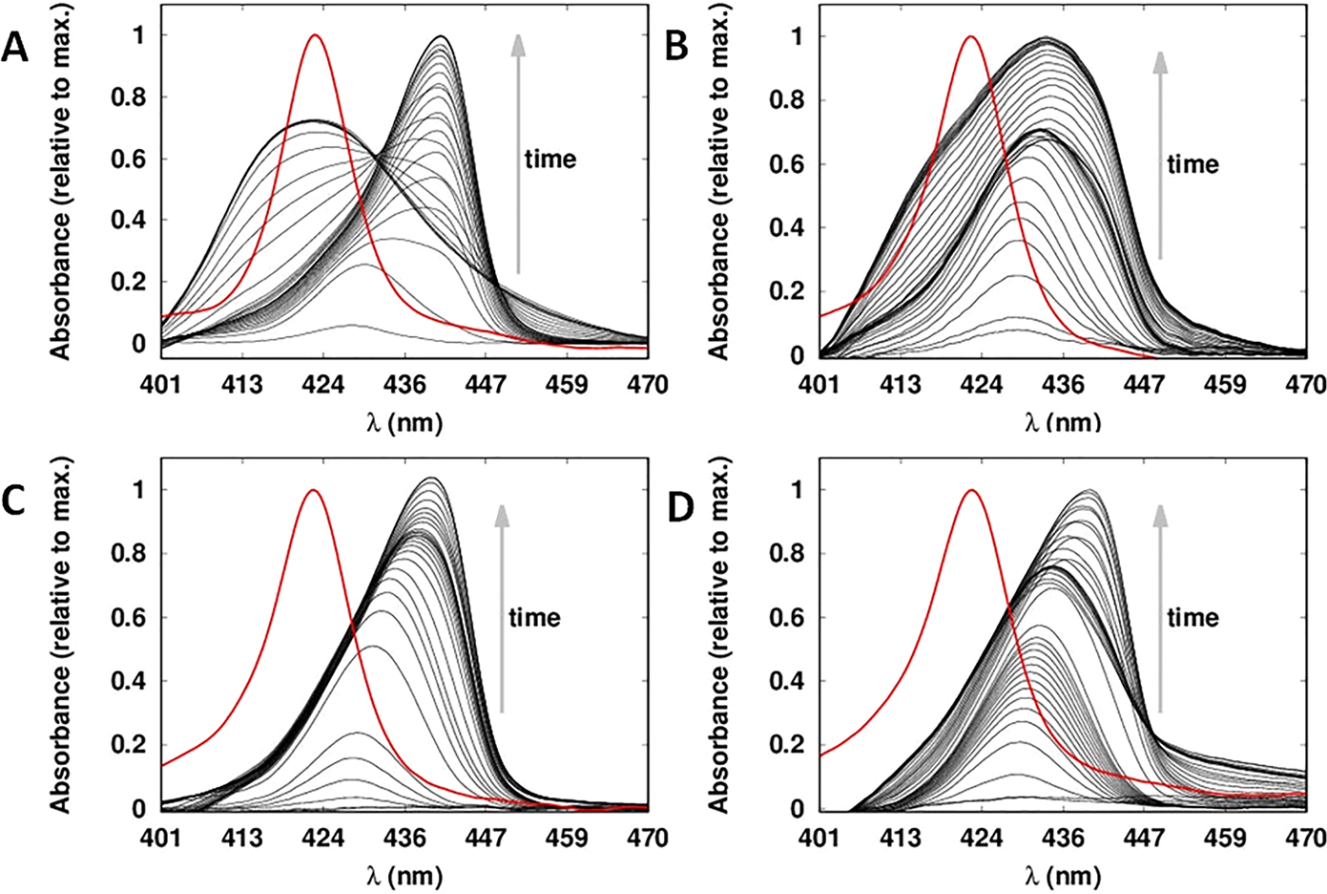
Modifying pathway selection to favor the formation of the metastable J-type nanostructure. Comparison of time-dependent TIR-UV–vis spectra of ZnTPPc interfacial self-assembly at the aqueous–organic interface as a function of the bulk aqueous citric acid concentration. [ZnTPPc]_aq_ was 8 μM, the pH was adjusted to 5.8, and the citric acid concentration was either (A) 10, (B) 50, (C) 100 or (D) 250 mM, respectively. The red spectra are that of bulk aqueous ZnTPPc at pH 5.8.

According to the Hofmeister series, citrate (and its derivatives) is a kosmotropic agent.^53^ Thus, in an effort to direct the pathway selection, we investigated how increasing the concentration of this supramolecular structure-stabilizing molecule in the bulk aqueous phase would influence the competing pathways. The evolution of the TIR-UV–vis spectra at pH 5.8, with a Γ_[ZnTPPc]_ of 4 nmol·cm^–2^ and employing 10, 50, 100, and 250 mM citric acid concentrations in the bulk aqueous phase, respectively, is shown in Figure 6. Under these conditions, the spectral evolution differs significantly with 10 mM citric acid in the bulk aqueous phase (as shown also in Figure 1A) under otherwise identical experimental conditions. At a concentration of 50 mM citric acid (Figure 6B), the ZnTPPc monomers initially adsorbed at the liquid–liquid interface and subsequently the Soret band red-shifted, indicating the formation of a J-type nanostructure (λ_max_ = 436 nm). Finally, a shoulder appeared centered at 418 nm and caused the main peak to blue-shift slightly by 2 nm. The latter suggests the presence of both interfacial nanostructures, with the J-type predominant over the H-type. An analysis by PCA was performed (Figure S13). However, due to severe overlapping of the spectra, only one significant component was detected in this data set. At citric acid concentrations ≥100 mM, the band (or shoulder) corresponding to the H-type nanostructure (λ_max_ = 418 nm) disappeared, and only red-shifted spectra were observed (λ_max_ = 442 nm), see Figure 6B–D. These TIR-UV–vis spectra remained unchanged over a period of 24 h. Thus, we concluded that at high citric acid concentrations, formation of the H-type nanostructure was completely inhibited.

**Figure 7:**
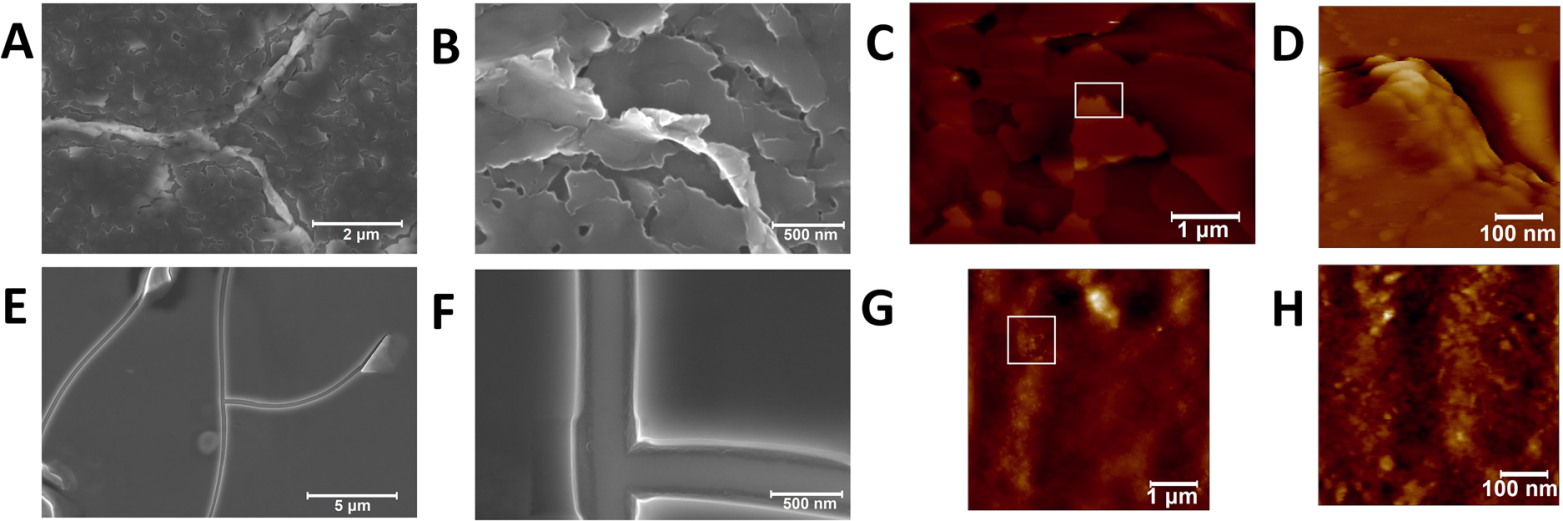
*Ex situ* characterization of the morphology of the interfacial ZnTPPc films by SEM and AFM. The interfacial ZnTPPc films were prepared with either (A–D) 10 mM or (E, F) 100 mM bulk aqueous citric acid concentrations, leading to predominately H- or J-type nanostructures in the films, respectively. Otherwise the experimental conditions were identical, as described in Figure 6. (D, H) AFM images recorded using semicontact mode of the areas of the films indicated by the white rectangles in (C, G), respectively.

The microscopic morphologies of the films of interfacial ZnTPPc nanostructures self-assembled at pH 5.8, with a Γ_[ZnTPPc]_ of 4 nmol·cm^–2^ and using either 10 or 100 mM citric acid in the bulk aqueous phase, were probed *ex situ* using scanning electron microscopy (SEM) and AFM (Figure 7). The influence of the citric acid concentration on the microscopic morphologies was profound, with 10 mM citric acid leading to the H-type nanostructures predominantly and 100 mM citric acid leading to the J-type nanostructures exclusively. Both SEM (Figure 7A,B) and AFM (Figure 7C,D) images clearly show that films consisting of flakes, some of which were stacked over each other, were formed using 10 mM citric acid. By contrast, films that were largely planar and without flakes were formed using 100 mM citric acid (Figure 7E–H). Furthermore, the presence of flakes significantly increased the root-mean-square (RMS) roughness of the films formed using 10 mM citric acid compared with the planar films formed using 100 mM citric acid, as measured by AFM and summarized in Table S7.

## Conclusions

Our kinetic analysis of the ZnTPPc self-assembly process using TIR-UV–vis spectra obtained *in situ* at the liquid–liquid interface showed the presence of kinetically favored metastable J-type nanostructures that form quickly but then transform into the thermodynamically favored H-type nanostructures. Numerical modeling of the kinetic data suggests that both nanostructures were produced by a cooperative (nucleation–elongation) mechanism. These nanostructures formed in parallel and competed for the free monomers adsorbed at the interface. Upon confirming that spontaneous supramolecular polymerization of ZnTPPc at the liquid–liquid interface is indeed controlled by pathway complexity, we demonstrated that varying the concentration of the kosmotropic citric acid aqueous electrolyte can change the thermodynamic preference of the assembly process. We can force aggregation completely down the kinetically favored pathway so that, by increasing the concentration of citric acid, we obtain only metastable J-type nanostructures. We show that the morphology of the resulting interfacial films of ZnTPPc nanostructures is significantly altered by the citric acid concentration using *ex situ* AFM and SEM analysis.

This work demonstrates that the stability of supramolecular materials can be manipulated in a controllable fashion at an immiscible liquid–liquid interface. Such pathway selection opens opportunities to rationally design optimal nanostructures from the same building blocks with different targeted features for specific applications, such as in photovoltaic^54^ and molecular electronic^55,56^ technologies. Furthermore, the presence of competing self-assembly pathways at liquid–liquid interfaces is not restricted to porphyrins and should be readily observed in other systems, for example, the formation of natural protein-based fibrils on membranes.^57,58^

## Supporting Information Available

Experimental and computational methods. Supplementary text detailing both models used to simulate the kinetic data, and details of MCMC method. Supplementary figures and tables describing the experimental methodology, UV–vis spectroscopic studies probing the effects of pH and porphyrin concentration on the kinetics of interfacial nanostructure formation, and PCA and MCMC analysis of the kinetic data sets (PDF)
