# Supplementary material for: Pathway Complexity in Supramolecular Porphyrin Self-Assembly at an Immiscible Liquid–Liquid Interface
Source: J Am Chem Soc. 2021 Jun 11;143(24):9060–9. doi: 10.1021/jacs.1c02481 (PMC8227452; doi:10.1021/jacs.1c02481)
Supplement: Supplementary file 1 — ja1c02481_si_001.pdf [file ja1c02481_si_001.pdf]

Supporting Information

Pathway Complexity in Supramolecular Porphyrin Self-Assembly at an Immiscible Liquid|Liquid Interface

Iván Robayo-Molina,[a] Andrés F. Molina-Osorio,[a] Luke Guinane,[b] Syed A.M. Tofail[b] and Micheál D. Scanlon*[a,c]

[a] The Bernal Institute and Department of Chemical Sciences, School of Natural Sciences, University of Limerick (UL), Limerick V94 T9PX, Ireland.

[b] The Bernal Institute and Department of Physics, School of Natural Sciences, University of Limerick (UL), Limerick V94 T9PX, Ireland.

[c] Advanced Materials and Bioengineering (AMBER) Centre, , CRANN Institute, Trinity College Dublin (TCD), Dublin 2, D02 PN40, Ireland.

*Corresponding author (M.D. Scanlon). E-mail: micheal.scanlon@ul.ie

TABLE OF CONTENTS	
**S1.** Experimental and computational methods.	**S3**
**S2**. Model 1: coupling of two competitive cooperative (nucleation-elongation) pathways.	**S5**
**S3.** Model 2: coupling of competitive isodesmic and cooperative pathways.	**S7**
**S4.** The Markov Chain Monte Carlo (MCMC) fitting procedure.	**S10**
**S5.** Supplementary figures.	**S11**
**Figure S1.** Experimental methodologies.	**S11**
**Figure S2.** Baseline correction.	**S12**
**Figure S3.** The influence of the aqueous phase pH on ZnTPPc interfacial self-assembly.	**S13**
**Figure S4.** The influence of the porphyrin concentration on ZnTPPc interfacial self-assembly.	**S14**
**Figure S5.** Scree plot and representation of the PCA results for the TIR-UV/vis dataset reported in Figure 1A (main text).	**S15**
**Figure S6.** Multivariate Curve Resolution–Alternating Least Squares (MCR-ALS) analysis of the kinetics of interfacial ZnTPPc self-assembly.	**S16**
**Table S1.** Quality control parameters extracted from MCR-ALS algorithms for each dataset.	**S17**
**Table S2.** Number of accepted and rejected steps, and number of iterations the covariance was updated for the MCMC run.	**S17**
**Figure S7.** MCMC traces of the parameters from kinetic Model 1.	**S17**
**Figure S8.** MCMC traces of the parameters from kinetic Model 2.	**S18**
**Figure S9.** Histogram of the parameter distributions for kinetic Model 1.	**S19**
**Table S3.** Parameter distributions obtained from the MCMC for kinetic Model 1.	**S20**
**Figure S10.** Histogram of the parameter distributions for kinetic Model 2.	**S20**
**Table S4.** Parameter distribution obtained from the MCMC for kinetic Model 2.	**S21**
**Table S5.** Normalized sensitivity coefficients for kinetic Model 1.	**S22**
**Table S6.** Normalized sensitivity coefficients for kinetic Model 2.	**S22**
**Figure S11.** Sensitivity coefficients as a function of time for kinetic Model 1 for **(A)** the J-type nanostructure and **(B)** the H-type nanostructure.	**S23**
**Figure S12.** Sensitivity coefficients as a function of time for kinetic Model 2 for **(A)** the J-type nanostructure and **(B)** the H-type nanostructure.	**S23**
**Figure S13.** Scree plot and representation of the PCA results for the TIR-UV/vis dataset reported in Figure 6B (main text).	**S24**
**Table S7.** Summary of AFM parameters	**S25**
**S5.** Supplementary References.	**S26**

S1. EXPERIMENTAL AND COMPUTATIONAL METHODS

Reagents. All chemicals were used as received without further purification. All aqueous solutions were prepared using high purity water (>18.2 MΩ·cm) from a Millipore MilliQ filtration system. α,α,α-trifluorotoluene (TFT, ≥99%) , lithium hydroxide monohydrate ($LiOH.H_2O$, ≥98%), methyltrichlorosilane (99%) and citric acid (≥99.5%) were purchased from Sigma-Aldrich. Zinc(II) 5,10,15,20-(tetra-4-carboxyphenyl)porphyrin (ZnTPPc) was obtained from PorphyChem. Aqueous solutions of ZnTPPc were prepared by directly dissolving the solid in the lithium citrate buffer pre-adjusted to the desired pH, followed by sonication of the solution for three minutes.

In situ **UV/vis spectroscopy in total internal reflection (TIR-UV/vis).** ZnTPPc self-assembly was studied *in situ* at the interface between an aqueous phase containing 10 mM (analytical concentration) citric acid and a neat organic phase (TFT) by TIR-UV/vis using a custom-built optical setup (Figure S1). Spectra were obtained every 0.5 s for up to 1000 s. The self-assembly process was performed using the "Porphyrin Last" protocol,[1] involving the addition of a known amount of porphyrin stock solution to the aqueous phase. The interfacial ZnTPPc concentration ($\Gamma_{[\mathrm{ZnTPPc}]}$) was determined using the isotherm of this biphasic system reported recently.[2] The pH of the aqueous phase was adjusted to values between 5.0 and 6.8 with LiOH, whereas the bulk aqueous ZnTPPc concentration ($[\mathrm{ZnTPPc}]_{\mathrm{aq.}}$) was 8 μM, *i.e.*, equivalent to a $\Gamma_{[\mathrm{ZnTPPc}]}$ value of 4 nmol·cm^{-2} at pH 5.8, unless stated otherwise.

Ex situ **microscopy characterisation.** The porphyrin films were gently transferred to a silicon substrate for SEM and AFM analysis by bringing the solid support into contact with the interface. Prior to imaging, the samples were sequentially rinsed with water and TFT. AFM was performed using NT-MDT's Ntegra Spectra II. The topography was recorded using semi-contact mode. The radius of curvature of the probe tip was less than 35 nm. The resonant frequency of the probe was 134.63 kHz. The probe stiffness was 5.83 N·m^{-1}. The gain of the lock-in-amplifier was set to 0.4. The scan size was set to 256 × 256 pixels. As the samples were expected to be rough in nature due to the stacking of flakes, the scan rate was 0.5 Hz.

Multivariate Curve Resolution–Alternating Least Squares (MCR-ALS) analysis. MCR-ALS was used to analyze the spectral evolution of interfacial ZnTPPc at the aqueous|organic interface. This tool allows the spectral evolution of individual H- and J-type nanostructures to be separated. The calculations described herein were performed in *R* (the foundation for statistical computers, version 3.4.4).[3] All UV/vis spectra measured during the

self-assembly process were arranged in a data matrix $Y(r \times c)$, where rows r were the spectra recorded at different times during the reaction and columns c were kinetic profiles collected at different wavelengths. The raw spectra were further treated using the package baseline[4] for smoothing and correcting the drift of the signal (Figure S2).

The decomposition of the matrix Y was achieved by using MCR-ALS, a well-established and widespread decomposition method meant to solve complex mixtures without any assumptions about the composition of the system.[5] Importantly, significant chemical information can be introduced in the optimization process under the form of constraints.[6,7] MCR-ALS decomposes the matrix Y according to the following equation

$$Y = CS^T + E \qquad \text{(S1)}$$

where matrix S^T ($n \times c$) contains the spectral profiles of n pure resolved components, matrix C ($r \times n$) describes the concentration profiles of these n species, and E represents the error matrix associated with the reconstruction. The first step of the decomposition required the determination of the number of significant components in the experimental data matrix by Principal Component Analysis (PCA). PCA is a reduction tool designed to identify the amount of variance present in a certain dataset and the determination of linearly independent components. Therefore, PCA allows the rank reduction of a large dataset to a few relevant components. This tool was implemented using the package FactoMineR[8] from R.

MCR-ALS was used according to the functions in package ALS.[9] For MCR, only the evolution of the porphyrin Soret band was analysed in the wavelength range from 400 to 484 nm. The initial matrix C was estimated by means of the detection of the purest concentration profiles[10] with a selected level of noise of 5%. The ALS routine was run employing the following soft constraints: non-negativity and unimodality for both pure spectra and concentration profiles. The “badness” of fit of the model obtained by MCR-ALS was evaluated by determining the Lack of Fit (LOF) parameter using the following equation:

$$\%\mathrm{LOF} = 100 \times \sqrt{\frac{\sum_i^r \sum_j^c \left(y_{ij}^*\right)^2}{\sum_i^r \sum_j^c y_{ij}^2}} \qquad \text{(S2)}$$

where y_{ij} and y_{ij}^* are the experimental and calculated absorbance values, respectively. A second parameter was used to corroborate the quality of the optimization; the percentage of explained variance (r^2), defined as

$$r^2 = 100 \times \frac{\sum_i^r \sum_j^c \left(y_{ij}^*\right)^2}{\sum_i^r \sum_j^c y_{ij}^2} \qquad \text{(S3)}$$

S2. MODEL 1: COUPLING OF TWO COMPETITIVE COOPERATIVE (NUCLEATION-ELONGATION) PATHWAYS

The interfacial self-assembly of ZnTPPc can be explored using a one-dimensional nanostructure formation (or aggregation). This means we only consider the aggregation of a chain, and interactions with other chains in two- or three-dimensions are negligible. We modelled the size of the nanostructures by monomer association and dissociation, hence the proposed mechanism for the *cooperative pathway (J-type nanostructure)* is given by:

$$\begin{gathered} M + M \rightleftharpoons_{k_2}^{k_1} J_2 \\ M + J_2 \rightleftharpoons_{k_3}^{k_1} J_3 \\ M + J_3 \rightleftharpoons_{k_3}^{k_1} J_4 \\ M + J_4 \rightleftharpoons_{k_3}^{k_1} J_5 \\ M + J_5 \rightleftharpoons_{k_3}^{k_1} J_6 \\ M + J_6 \rightleftharpoons_{k_3}^{k_1} J_p \\ M + J_p \rightleftharpoons_{k_3}^{k_1} 2J_p \end{gathered} \qquad \text{(S4)}$$

The proposed mechanism for the other *cooperative pathway (H-type nanostructure)* is given by:

$$\begin{gathered} M + M \rightleftharpoons_{k_5}^{k_4} H_2 \\ M + H_2 \rightleftharpoons_{k_6}^{k_4} H_3 \\ M + H_3 \rightleftharpoons_{k_6}^{k_4} H_4 \\ M + H_4 \rightleftharpoons_{k_6}^{k_4} H_5 \\ M + H_5 \rightleftharpoons_{k_6}^{k_4} H_6 \\ M + H_6 \rightleftharpoons_{k_6}^{k_4} H_p \end{gathered} \qquad \text{(S5)}$$

Here, M is the monomeric ZnTPPc adsorbed at the immiscible liquid|liquid interface, and J_i and H_i are the J- and H-type nanostructures, respectively. These nanostructures consist

of i monomers. Nanostructures below a nucleus size n grow with a nucleation equilibrium constant K_n, described by the kinetic constants k_1, k_2, k_4 and k_5. The association constants are k_1 and k_4 and the dissociation constants are k_2 and k_5. Meanwhile, nanostructures equal to, or above, the nucleus size grow with an elongation equilibrium constant K_e, described by the kinetics constants k_1, k_3, k_4 and k_6. For cooperative polymerization, we have the following condition, $K_n < K_e$.

To reduce the computational cost, the length of the polymers was set to 7. Further increasing the size of the chain did not affect the output. In the cooperative pathway, the last step is an autocatalytic process and therefore, to fulfil the mass balance, step 6 is irreversible following the model of Frieden for the polymerization of actin.[11]

This mechanism produced a set of 13 ordinary differential equations (ODEs) that must be solved simultaneously.

$$\frac{dJ_2}{dt} = k_1([M]^2 - [M][J_3]) - k_2[J_2] + k_3[J_3]$$

$$\frac{dJ_i}{dt} = k_1[M]([J_{i-1}] - [J_i]) + k_3([J_{i+1}] - [J_i]) \quad 3 \leq i \leq 5$$

$$\frac{dJ_6}{dt} = k_1[M]([J_5] - [J_6]) + k_3[J_6]$$

$$\frac{dJ_p}{dt} = k_1[M]\big([J_6] - [J_p]\big) + k_3[J_6]$$

$$\frac{dH_2}{dt} = k_4([M]^2 - [M][H_3]) - k_5[H_2] + k_6[H_3]$$

$$\frac{dH_i}{dt} = k_4[M]([H_{i-1}] - [H_i]) + k_6([H_{i+1}] - [H_i]) \quad 3 \leq i \leq 5$$

$$\frac{dH_6}{dt} = k_4[M]([H_5] - [H_6]) + k_6[H_6]$$

$$\frac{dH_p}{dt} = k_4[M]\big([H_6] - [H_p]\big) + k_6[H_6]$$

$$\frac{dM}{dt} = 2(k_2[J_2] - k_1[M]^2) - k_1[M]\sum_{i=3}^{7}[J_i] + k_3\sum_{i=3}^{6}[J_i] + k_3[J_p]^2 + 2(k_4[H_2] - k_5[M]^2) - k_4[M]\sum_{j=3}^{7}[H_j] + k_6\sum_{j=3}^{6}[H_j] + k_6[H_p]^2 \quad \text{(S6)}$$

The initial conditions (numbers at $t = 0$) are as follows:

$$[M](0) = M_0$$
$$[J_i](0) = 0$$
$$[H_j](0) = 0$$
(S7)

The following constraints are applied:

$$k_3[J_p] = 0.0$$
$$k_6[H_p] = 0.0$$
$$k_1/k_2 > 1$$
$$k_3/k_5 > 1$$
$$k_3/k_4 < 1$$
(S8)

The mass balance of the reaction is:

$$M_0 = M + i\sum_{i=2}^{n} J_i + J_p + j\sum_{j=2}^{6} H_j + H_p$$
(S9)

Finally, the signals that are observed are assumed to originate from ZnTPPc in the monomeric state, in the J-type nanostructure and in the H-type nanostructure, the concentrations of which are given by [J_{agg}] and [H_{agg}]:

$$[J_{agg}] = [J_p]$$
$$[H_{agg}] = [H_p]$$
(S10)

The corresponding system of coupled ODEs is solved using the functions provided by the **deSolve** package in **R**.[12] An interactive version of this model can be found in the following link https://entropia88.shinyapps.io/Shiny/

S3. MODEL 2: COUPLING OF COMPETITIVE ISODESMIC AND COOPERATIVE PATHWAYS

In this model, the interfacial self-assembly of ZnTPPc is explained by means of isodesmic and cooperative pathways. The *isodesmic pathway* is given by:

$$M + M \rightleftharpoons_{k_2}^{k_1} J_2$$

$$M + J_2 \rightleftharpoons_{k_2}^{k_1} J_3$$

$$M + J_3 \rightleftharpoons_{k_2}^{k_1} J_4$$

$$M + J_4 \rightleftharpoons_{k_2}^{k_1} J_5$$

$$M + J_5 \rightleftharpoons_{k_2}^{k_1} J_6$$

$$M + J_6 \rightleftharpoons_{k_2}^{k_1} J_7$$

(S11)

The *cooperative pathway* is given by:

$$M + M \rightleftharpoons_{k_4}^{k_3} H_2$$

$$M + H_2 \rightleftharpoons_{k_4}^{k_3} H_3$$

$$M + H_3 \rightleftharpoons_{k_5}^{k_3} H_4$$

$$M + H_4 \rightleftharpoons_{k_5}^{k_3} H_5$$

$$M + H_5 \rightleftharpoons_{k_5}^{k_3} H_6$$

$$M + H_6 \rightleftharpoons_{k_5}^{k_3} H_p$$

$$M + H_p \rightleftharpoons_{k_5}^{k_3} 2H_p$$

(S12)

Here, M is the monomeric ZnTPPc adsorbed at the immiscible liquid|liquid interface, and J_i and H_i are the J-type and H-type nanostructures, respectively. These nanostructures consist of i monomers. For the isodesmic pathway, the association and dissociation constants are k_1 and k_2, respectively. For the cooperative pathway, nanostructures below a nucleus size n grow with a nucleation equilibrium constant K_n, described by the kinetic constants k_3 and k_4 for the association and dissociation, respectively. Meanwhile, nanostructures equal to or above the nucleus size grow with an elongation equilibrium constant K_e, described by the kinetic constants k_3 and k_5. For cooperative polymerization, $K_n < K_e$.

To reduce the computational cost, the length of the polymers was set to 7. Further increases of the size of the chain did not affect the output. In the cooperative pathway, the last

step is an autocatalytic process. Therefore, to fulfil the mass balance, step 6 is irreversible, following the model of Frieden for polymerization of actin.[11]

This mechanism produced a set of 13 ODEs that must be solved simultaneously.

$$\frac{dJ_i}{dt} = k_1[M]([J_{i-1}] - [J_i]) + k_2([J_{i+1}] - [J_i]) \quad i \geq 2$$

$$\frac{dJ_n}{dt} = k_1[M][J_{n-1}] + k_2[J_n]$$

$$\frac{dH_2}{dt} = k_3([M]^2 - [M][H_3]) - k_4[H_2] + k_5[H_5]$$

$$\frac{dH_i}{dt} = k_3[M]([H_{i-1}] - [H_i]) + k_5([H_{i+1}] - [H_i]) \quad 3 \leq i \leq 5$$

$$\frac{dH_6}{dt} = k_3[M]([H_5] - [H_6]) + k_5[H_6]$$

$$\frac{dH_p}{dt} = k_3[M]\big([H_6] - [H_p]\big) + k_5[H_6]$$

$$\frac{dM}{dt} = 2(k_2[J_2] - k_1[M]^2) - k_1[M]^2\sum_{i=3}^{7}[J_i] + k_3\sum_{i=3}^{6}[J_i] + 2(k_4[H_2] - k_3[M]^2) - k_3[M]\sum_{j=3}^{7}[H_j] + k_5\sum_{j=3}^{6}[H_j] + k_5[H_p]^2$$

(S13)

The initial conditions (numbers at $t = 0$) are as follows:

$$[M](0) = M_0$$

$$[J_i](0) = 0$$

$$[H_j](0) = 0$$

(S14)

The following constraints are applied:

$$k_5[H_p] = 0.0$$

$$k_1/k_2 > 1$$

$$k_3/k_5 > 1$$

$$k_3/k_4 < 1$$

(S15)

The mass balance of the reaction is:

$$M_0 = M + i\sum_{i=2}^{n} J_i + j\sum_{j=2}^{6} H_j + H_p$$

(S16)

Finally, the signals that are observed are assumed to originate from ZnTPPc in the monomeric state, in the J-type nanostructure and in the H-type nanostructure, the concentrations of which are given by $[J_{agg}]$ and $[H_{agg}]$:

$$[J_{agg}] = i\sum_{i=2}^{n}[J_i]$$

$$[H_{agg}] = [H_p]$$

(S17)

The corresponding system of coupled ODEs is solved using the functions provided by the **deSolve** package in **R.**[12] An interactive version of this model can be found at the following link https://entropia88.shinyapps.io/Shiny/

S4. THE MARKOV CHAIN MONTE CARLO (MCMC) FITTING PROCEDURE

The main issue affecting the resolution of bilinear data in MCR is the non-unicity of the solution,[13] due to rotational and intensity ambiguities of the solution. Intensity ambiguities are related to scaling issues and can be solved by the normalization of concentration profiles or resolved spectra. Rotational ambiguities are related to changes in the shape of the sought profiles, and this type of ambiguity is the most problematic for robustness and interpretation of MCR results. The use of constraints can diminish the rotational ambiguity, but not eliminate it completely. Consequently, solutions in MCR are usually represented as feasible bands,[14] that is the space of possible solutions.

A regular nonlinear fitting procedure is not the most accurate algorithm considering these feasible bands. Thus, it is important to provide an estimate of the parameter uncertainty, and to quantify the effect of that uncertainty on the observed variables. For this purpose, the

method chosen was Markov Chain Monte Carlo (MCMC). MCMC was implemented using the package **FME**.[12] **FME** uses MCMC with the Delayed Rejection and Adaptive Metropolis procedure.[15] MCMC is an efficient sampling method where the selection of the next parameter combination depends on the last parameter set and the resulting deviation between the model and the observation. Therefore, sampling is concentrated in the region with high likelihood. This makes the method more efficient.

To avoid 'burn-in', the algorithm was started with the optimal parameter set as returned from the nonlinear fitting algorithm provided by the package **FME**. The parameter sets were taken using a Latin Hyperube Sampling method, with the Latinhyper function implemented in the FME package. MCMC was run with a number of 7000 steps, a delayed reaction of 2, the number of iterations after which the parameter covariance matrix is evaluated was set to 100, and a low variance weight was given to the prior distribution compared to the posterior distribution (wvar = 0.1). Additionally, the lower and upper bounds of the parameters were carefully selected to fulfil the conditions (S5) and (S12) in Models 1 and 2, respectively.

S5. SUPPLEMENTARY FIGURES

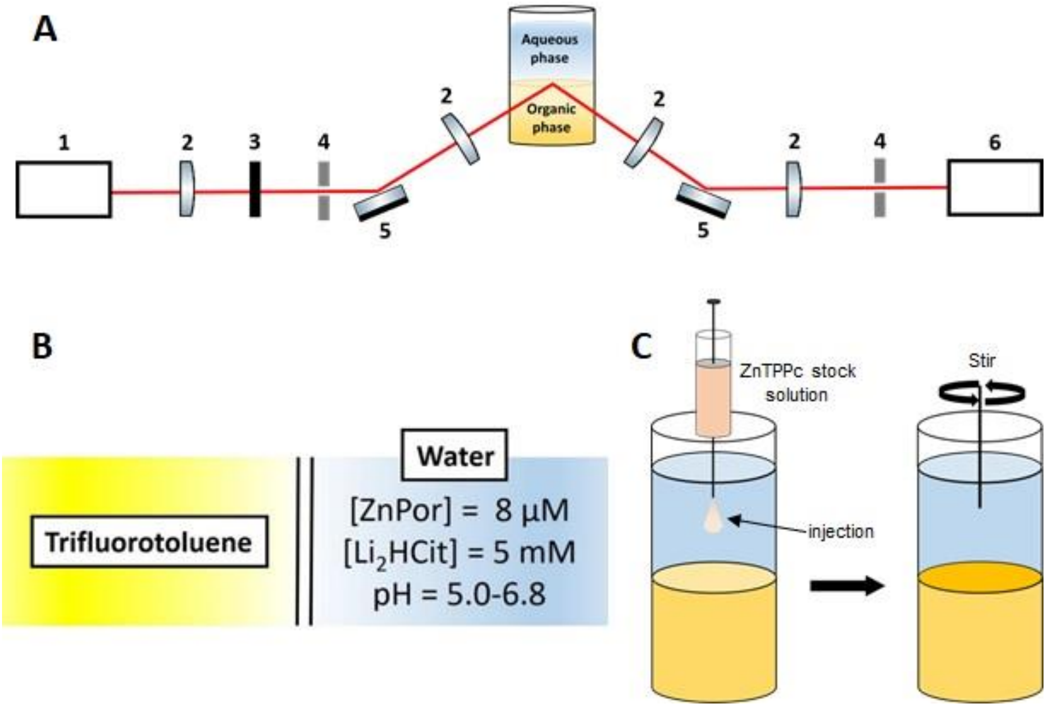

Figure S1. Experimental methodologies. (A) Schematic representation of the setup for TIR-UV/vis absorbance measurements in TIR mode. (1) Xe light source, (2) plano-convex lenses,

(3) infrared filter, (4) iris diaphragm, (5) mirror, and (6) Ocean Optics Maya2000 Pro spectrometer. The angle of incidence was set to 80º to ensure TIR conditions. **(B)** Schematic description of the biphasic system used during this work. The vertical double line represents the polarisable interface between the organic solvent α,α,α-trifluorotoluene (TFT) and the aqueous phase. **(C)** Simple depiction of the mixture protocol using the "Porphyrin Last" procedure. All vials were previously silanized by adding some drops of methyltrichlorosilane into the organic phase of the biphasic system. Once the interface is flat, the reaction was stopped by emptying the vials and washing them extensively with acetone.

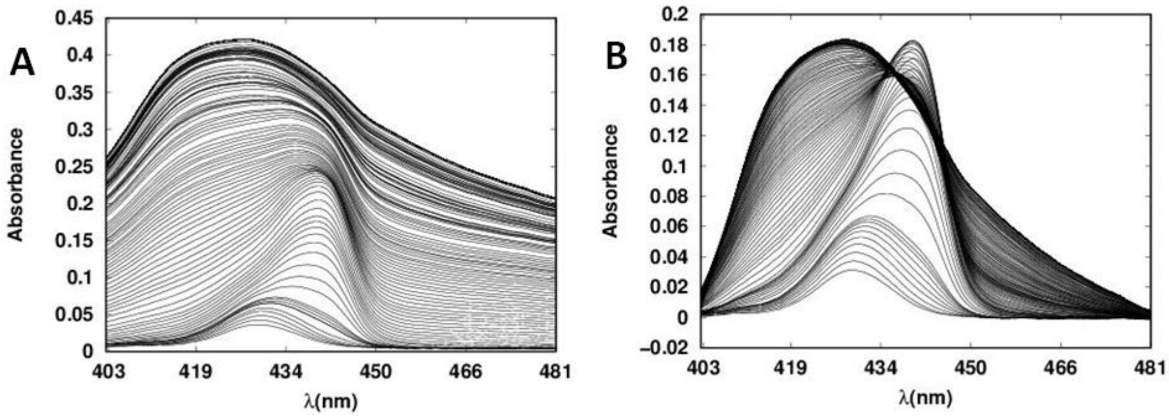

Figure S2. Baseline correction. (A) Example of unprocessed spectral data which clearly shows the drift of the signal. The raw spectra were further treated **(C)** using R (version 4.0.3) with the function *baseline.rfbaseline* for a Robust Baseline Estimation (a function that is included in the package baseline).[6]

ZnTPPc self-assembly at an immiscible aqueous|organic interface is very sensitive to the pH of the aqueous phase.[2,16] Above the pK_a value of the carboxyl group on the porphyrin (pK_a = 5.8),[9] the carboxyl groups are primarily deprotonated and the ZnTPPc monomers are negatively charged. Thus, the adsorbed monomers at the interface repel each other electrostatically and interfacial nanostructure self-assembly is inhibited. Consequently, only a band centred at 430 nm is detected in Figures S3D-F, corresponding to the Soret band of ZnTPPc monomers adsorbed at the interface. Below the pK_a, the ZnTPPc molecules aggregate uncontrollably in the bulk aqueous phase (red lines, Figure S1A-B). Consequently, the quantity of free monomers adsorbed at the interface is very limited, and interfacial nanostructure formation does not take place. Only at a pH value equals to the pK_a of the carboxyl groups can interfacial ZnTPPc self-assembly proceed (Figure S3C).

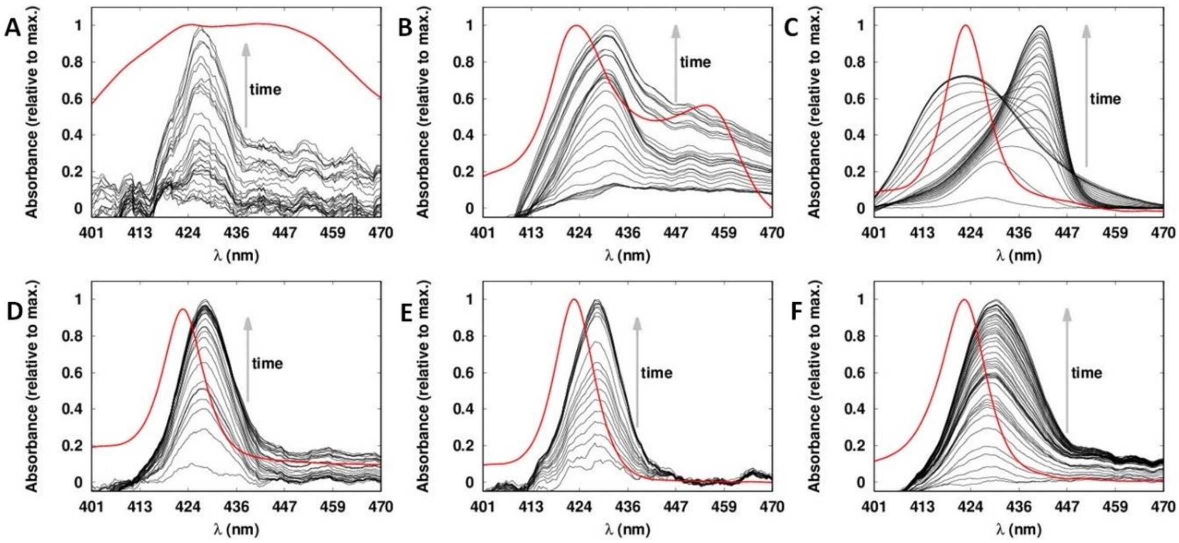

Figure S3. The influence of the aqueous phase pH on ZnTPPc interfacial self-assembly. Comparison of time dependent TIR-UV/vis spectra of ZnTPPc interfacial self-assembly when the aqueous phase pH was adjusted to **(A)** 5.0, **(B)** 5.5, **(C)** 5.8, **(D)** 6.1, **(E)** 6.5 and **(F)** 6.8. All other biphasic experimental conditions were identical (bulk aqueous ZnTPPc concentration of 8 μM, 10 mM citric acid aqueous electrolyte, and the organic phase was neat TFT). TIR-UV/vis spectra were taken every 0.5 s for 500 seconds. The red spectra are that of bulk aqueous ZnTPPc at each pH.

Below 5 μM [ZnTPPc]$_{aq.}$, the interfacial self-assembly process did not proceed, and only the Soret band attributed to adsorbed monomers at the aqueous|organic interface were detected (Figure S4A-B). For [ZnTPPc]$_{aq.}$ values between 5 and 10 μM, interfacial nanostructure formation proceeded, and all experimental datasets presented the same dynamic behaviour (Figure S4C-E). This dynamic behaviour is discussed in detail in the main text and illustrated in Figure 1C.

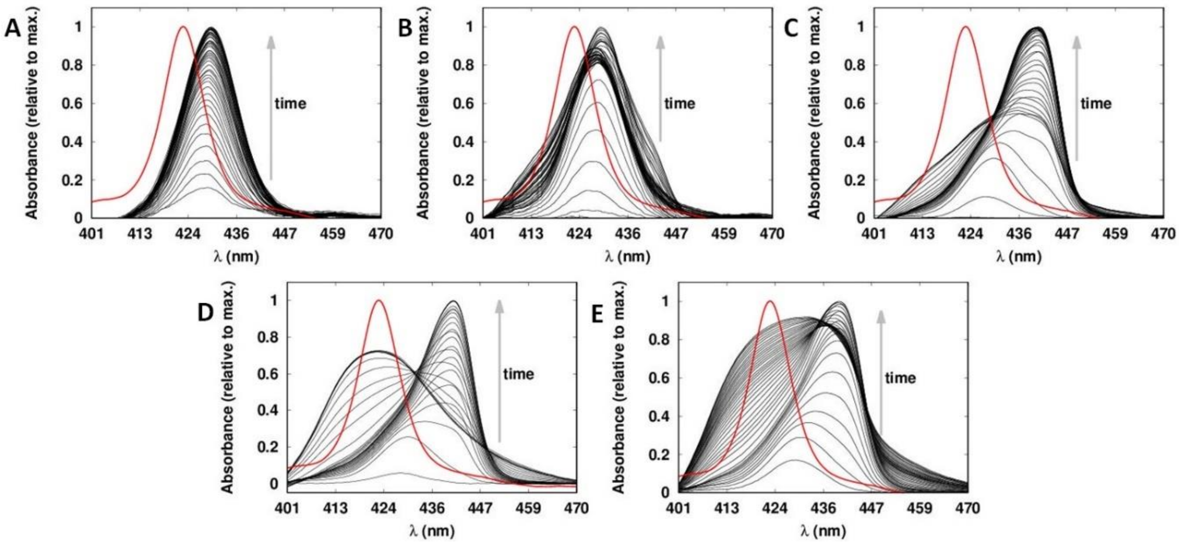

Figure S4. The influence of the porphyrin concentration on ZnTPPc interfacial self-assembly. Comparison of time dependent TIR-UV/vis spectra of ZnTPPc interfacial self-assembly when the bulk aqueous ZnTPPc concentration ([ZnTPPc]$_{aq.}$) was adjusted to **(A)** 1.0, **(B)** 2.0, **(C)** 5.0, **(D)** 8.0, and **(E)** 10.0 μM. All other biphasic experimental conditions were identical (aqueous pH of 5.8, 10 mM citric acid aqueous electrolyte, and the organic phase was neat TFT). TIR-UV/vis spectra were taken every 0.5 s for 500 seconds. The red spectra are that of bulk aqueous ZnTPPc at pH 5.8.

Figure S5A reports the scree plot for the TIR-UV/vis dataset in Figure 1A (main text; $\Gamma_{[ZnTPPc]}$ of 4 nmol·cm^{-2}). Eigenvalues associated to each component show an elbow in the second component, marking the separation between physiochemically meaningful components and noise-related ones. This result is confirmed by analyzing the scores and loadings from the PCA in Figure S5B-C. The scores and loadings appear noisier and unstructured from the third component. Based on these considerations, we ran the subsequent MCR-ALS analysis using two principal components (PCs, see Figure 2, main text). PC_1 was associated to an H-type nanostructure ($\lambda_{max.}$ = 418 nm) and the PC_2 to a J-type nanostructure ($\lambda_{max.}$ = 442 nm). The porphyrin monomers adsorbed at the interface were not a significant component that contributed to the variance of the dataset. Similar results were found for the other experimental datasets at $\Gamma_{[ZnTPPc]}$ values of 0.4 and 4.8 nmol·cm^{-2} (data not shown). The peak that appeared at 452 nm corresponds to an artefact generated by small distortions of the spectra obtained in Total Internal Reflection (TIR) Mode.

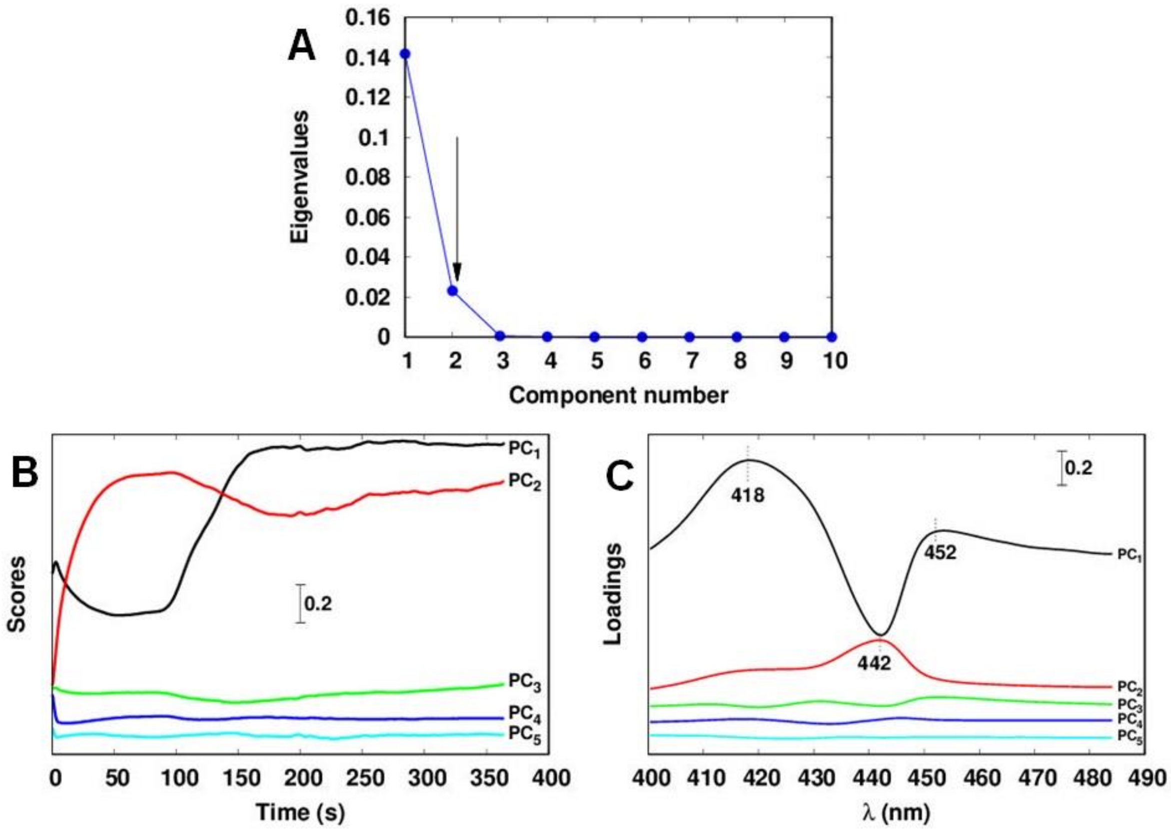

Figure S5. Scree plot and representation of the PCA results for the TIR-UV/vis dataset reported in Figure 1A (main text). (A) Scree plot, **(B)** PCA scores as function of time and **(C)** PCA loadings as a function of wavelength.

MCR successfully separated the original spectral data matrix intro the two types of nanostructures (Figure 2 main text and Figure S6). Based on the pure spectra obtained, one spectrum was assigned to a J-type nanostructure ($\lambda_{max.}$ = 442 nm) and the other to a H-type nanostructure ($\lambda_{max.}$ = 418 nm). The peak that appeared at 452 nm corresponds to an artefact generated by small distortions of the spectra obtained in TIR Mode, and the small variations of the H-type nanostructure are attributed to the interference of the monomer spectrum (λ_{max} 430 nm). Table S1 displays the quality parameters of the modelling, in all cases the % Lack of Fit (LOF) was close or below 5%, and the percentage of explained variance (r^2) was greater than 97%.

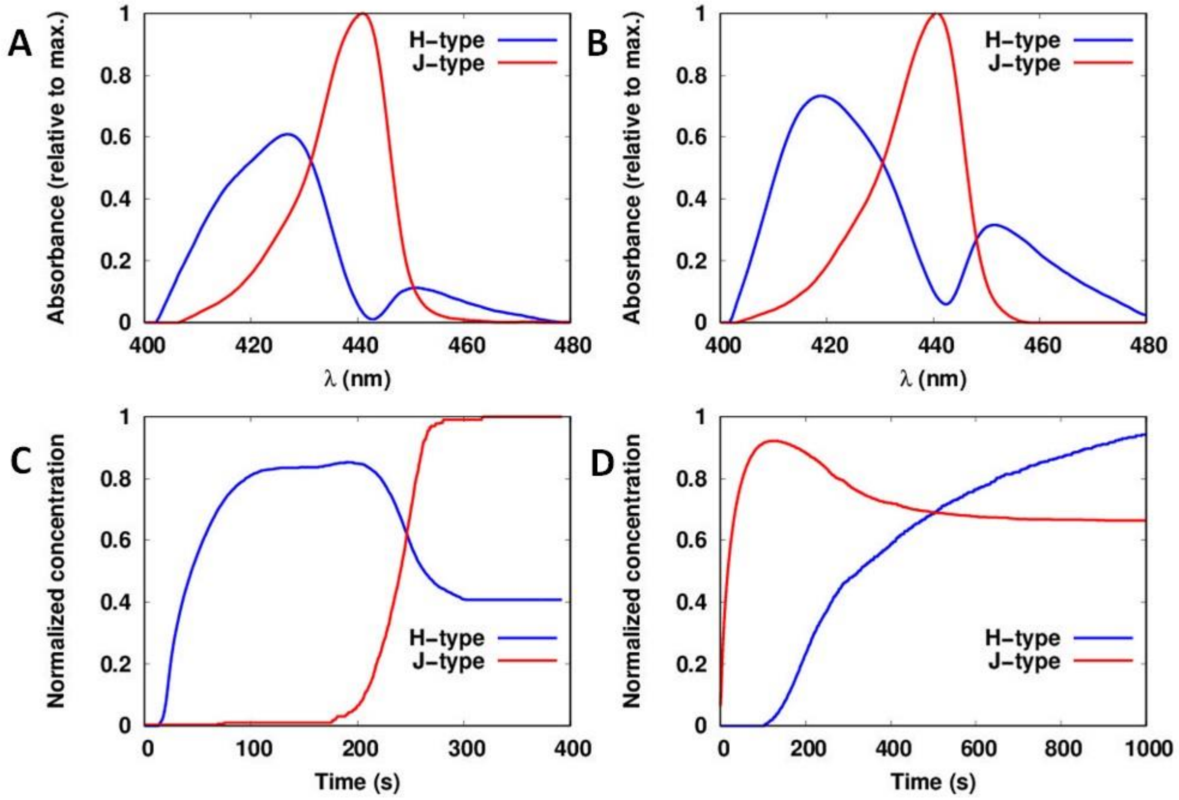

Figure S6. Multivariate Curve Resolution–Alternating Least Squares (MCR-ALS) analysis of the kinetics of interfacial ZnTPPc self-assembly. (A, B) MCR-ALS resolved the pure spectra of the H- and J-type nanostructures for interfacial ZnTPPc concentration ($\boldsymbol{\Gamma}_{[\mathbf{ZnTPPc}]}$) values of **(A)** 2.6 and **(B)** 4.8 nmol·cm^{-2} at pH 5.8, respectively. **(C, D)** The corresponding kinetic profiles for the H- and J-type nanostructures, $\boldsymbol{\Gamma}_{[\mathbf{ZnTPPc}]}$ values of **(C)** 2.6 and **(D)** 4.8 nmol·cm^{-2}, respectively.

Table S1. Quality control parameters extracted from MCR-ALS algorithms for each dataset.

$\Gamma_{[ZnTPPc]}$ (nmol·cm^{-2})	%LOF	Root sum squared (RSS)	r^2
2.6	1.97	0.2197	99.9
4.0	6.37	4.1581	97.2
4.8	2.56	2.3812	97.0

Table S2 shows the number of accepted and rejected steps for both models. Figures S7 and S8 show the traces of the MCMC chain (grey line) along the iterations for every parameter and the residuals. It is clearly seen that the chain converged because there is no apparent drift in each of the traces.

Table S2. Number of accepted and rejected steps, and number of iterations the covariance was updated for the MCMC run.

	Accepted steps	Rejected steps	Covariance update
**Model 1**	2843	5280	66
**Model 2**	1844	4251	69

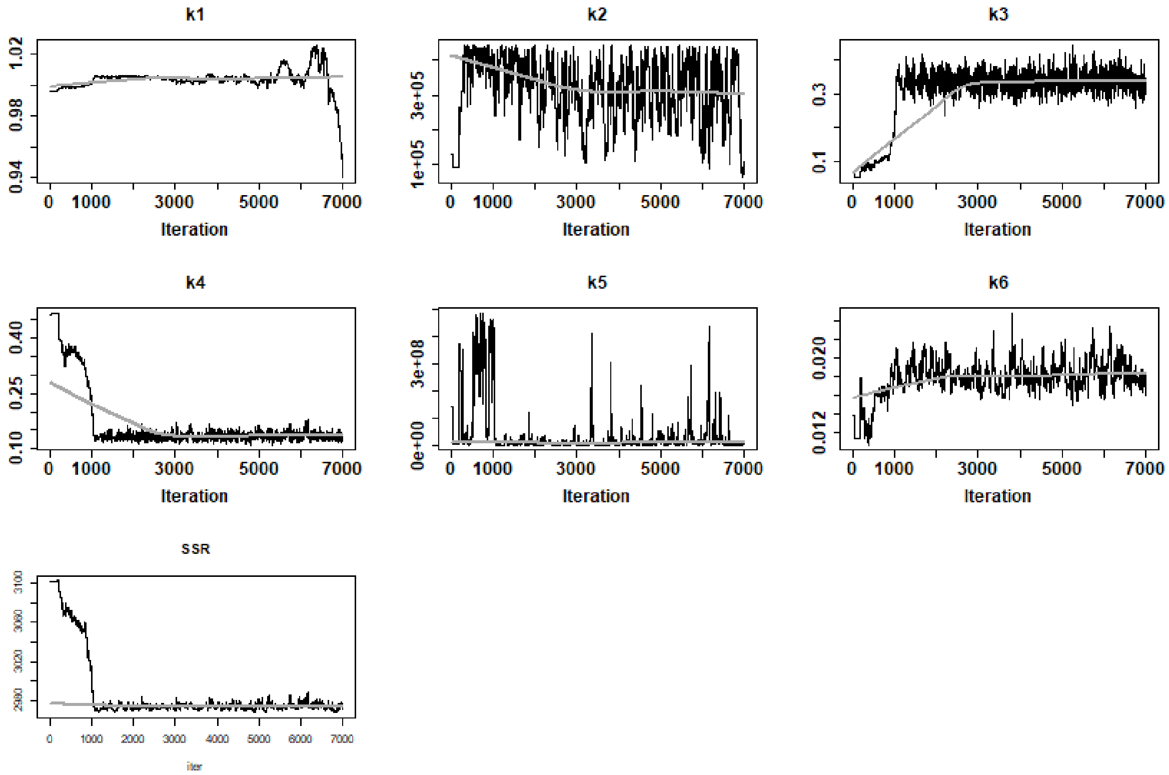

Figure S7. MCMC traces of the parameters from kinetic Model 1. SSR calculated is 1484.

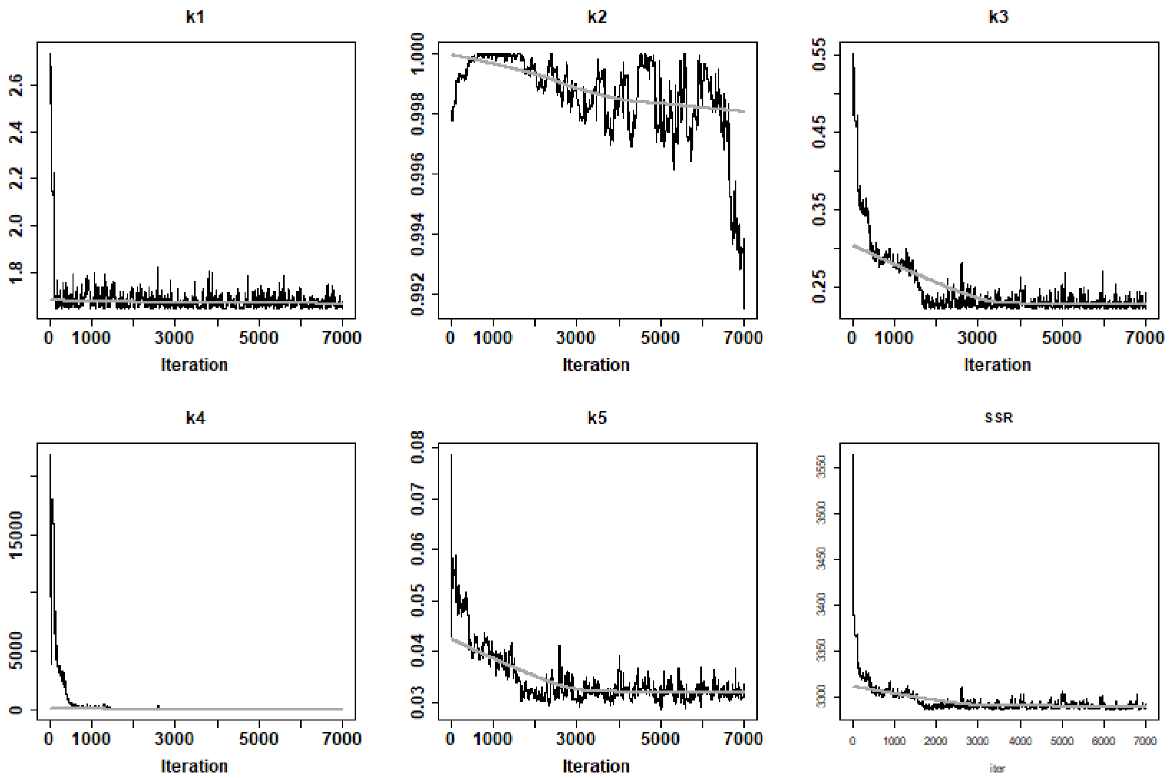

Figure S8. MCMC traces of the parameters from kinetic Model 2. SSR calculated is 1643.

The parameter distributions found by MCMC are plotted in Figures S9 and S10, and tabulated in Table S3 and S4, for Models 1 and 2, respectively. The dissociation constants of the nuclei for both models had a non-normal distribution, meaning that their values could change over a wide range, and this had no significant effect to the output. Hence, the uncertainty for these parameters was large. By contrast, the other parameters were normally distributed except for k_1 and k_3 in Model 2 which seem to have exponential distribution shapes. The latter may be due to the lower bound restriction imposed on these parameters to fulfil condition (S12).

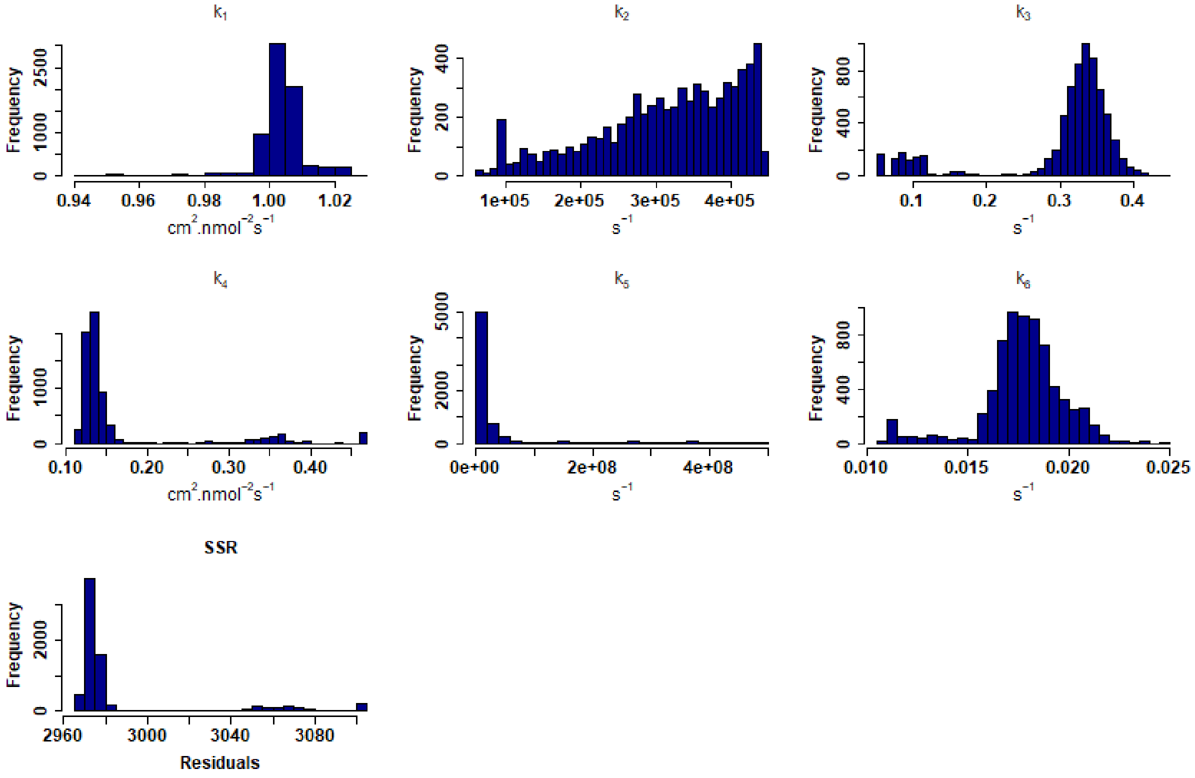

Figure S9. Histogram of the parameter distributions for kinetic Model 1.

Table S3. Parameter distributions obtained from the MCMC for kinetic Model 1.

	k_1 ($cm^2 \cdot nmol^{-1} \cdot s^{-1}$)	k_2 (s^{-1})	k_3 (s^{-1})	k_4 ($cm^2 \cdot nmol^{-1} \cdot s^{-1}$)	k_5 (s^{-1})	k_6 (s^{-1})
mean	1.003	3.15×10^5	3.02×10^{-1}	1.67×10^{-1}	4.57×10^7	1.77×10^{-2}
SD	7.80×10^{-3}	9.49×10^4	8.84×10^{-2}	8.56×10^{-2}	9.75×10^7	2.05×10^{-3}
Minimum value	9.40×10^{-1}	6.25×10^3	5.33×10^{-2}	1.10×10^{-1}	3.27×10^7	1.06×10^{-3}
Maximum value	1.03	4.42×10^5	4.43×10^{-1}	4.68×10^{-1}	4.8×10^8	2.45×10^{-2}
Q_1	1.002	2.57×10^5	3.07×10^{-1}	1.28×10^{-1}	4.83×10^6	1.69×10^{-2}
Q_2	1.004	3.32×10^5	3.31×10^{-1}	1.34×10^{-1}	8.48×10^6	1.78×10^{-2}
Q_3	1.006	3.95×10^4	3.50×10^{-1}	1.44×10^{-1}	2.43×10^7	1.88×10^{-2}

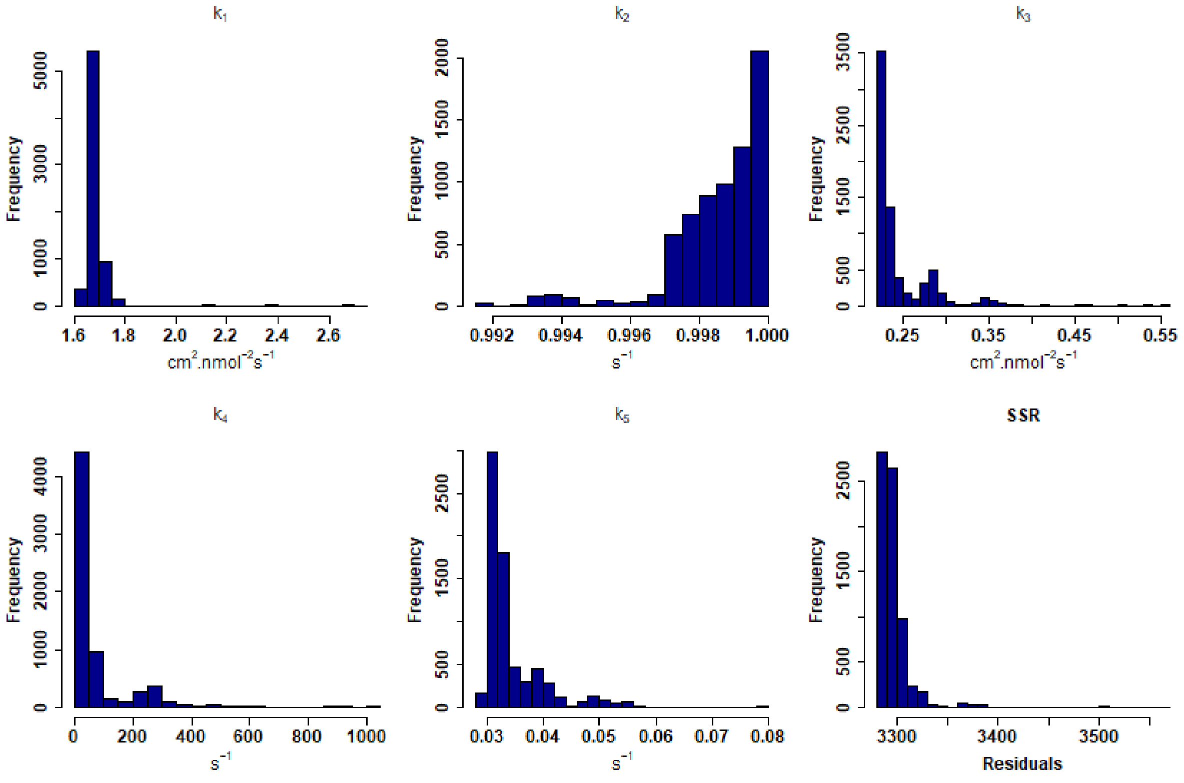

Figure S10. Histogram of the parameter distributions for kinetic Model 2.

Table S4. Parameter distribution obtained from the MCMC for kinetic Model 2.

	k_1 ($cm^2 \cdot nmol^{-1} \cdot s^{-1}$)	k_2 (s^{-1})	k_3 ($cm^2 \cdot nmol^{-1} \cdot s^{-1}$)	k_4 (s^{-1})	k_5 (s^{-1})
mean	1.69	9.99×10^{-1}	2.48×10^{-1}	4.20×10^{2}	$3.43x10^{-2}$
SD	9.29×10^{-2}	1.40×10^{-1}	4.42×10^{-2}	1.72×10^{3}	5.42×10^{-3}
Minimum value	1.64	9.92×10^{-1}	2.23×10^{-1}	18.0	2.85×10^{-2}
Maximum value	2.73	1.00	5.52×10^{-1}	2.19×10^{4}	7.88×10^{-2}
Q_1	1.66	9.98×10^{-1}	2.26×10^{-1}	3.24×10^{1}	3.13×10^{-2}
Q_2	1.67	9.99×10^{-1}	2.30×10^{-1}	4.02×10^{1}	3.22×10^{-2}
Q_3	1.69	1.00	2.49×10^{-1}	8.60×10^{1}	3.49×10^{-2}

To further investigate the parameter uncertainty found by MCMC, a sensitivity analysis was done. Local sensitivity analysis was performed according to the function provided by the package **FME**.[12] A matrix, S_{ij}, that contained the normalized and dimensionless sensitivities, was generated and every element of this matrix was defined as

$$\bar{S}_{ij} = \frac{\bar{k}_j}{\bar{y}_i} \cdot \frac{\partial y_i}{\partial k_j} \qquad \text{(S18)}$$

where y_i is an output variable, and k_j is the j_{th} parameter. These coefficients were normalized by the nominal value of y_i and k_j. The higher the absolute sensitivity value, the more important the parameter. Thus, the magnitudes of the sensitivity can be used to rank the importance of the parameter to the output variable.

A summary of these ranks for Models 1and 2 are presented in Tables S5 and S6. Here, L_1 and L_2 are defined as follows:

$$L_1 = \sum \frac{|S_{ij}|}{n} \qquad \text{(S19)}$$

$$L_2 = \sqrt{\frac{S_{ij}^2}{n}} \qquad \text{(S20)}$$

Table S5. Normalized sensitivity coefficients for kinetic Model 1.

	Value	L_1	L_2	Mean	Min	Max
k_1	9.83×10^{4}	1.91	3.39	-0.98	-10.76	11.11
k_2	2.65×10^{-5}	0.15	0.34	0.123	-1.00	1.00
k_3	3.21×10^{-1}	1.69	3.05	0.84	-3.60	10.16
k_4	1.30×10^{-1}	2.70	5.17	1.84	-5.16	16.38
k_5	4.17×10^{6}	0.10	0.23	0.044	-1.03	0.58
k_6	1.66×10^{-2}	0.31	0.40	0.076	-0.34	0.66

Table S6. Normalized sensitivity coefficients for kinetic Model 2.

	Value	L_1	L_2	Mean	Min	Max
k_1	1.65	3.05	4.30	-0.40	-11.20	7.20
k_2	1.00	2.68	3.60	0.13	-6.55	9.30
k_3	2.25×10^{-1}	3.08	4.50	0.98	-7.27	12.30
k_4	3.34×10^{1}	1.30	2.80	0.79	-3.08	9.20
k_5	3.20×10^{-2}	0.92	1.20	0.39	-0.68	2.20

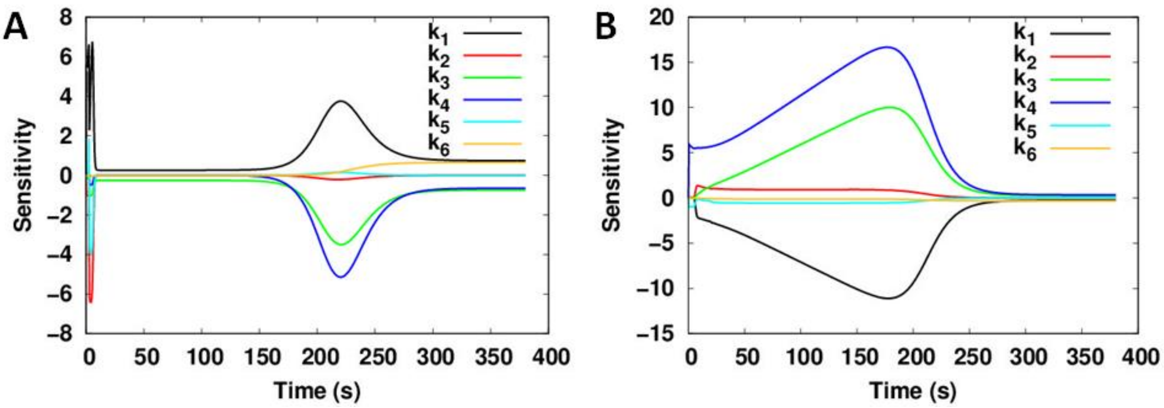

Figure S11. Sensitivity coefficients as a function of time for kinetic Model 1 for **(A)** the J-type nanostructure and **(B)** the H-type nanostructure. The $\Gamma_{[ZnTPPc]}$ value was 4.0 nmol·cm^{-2}.

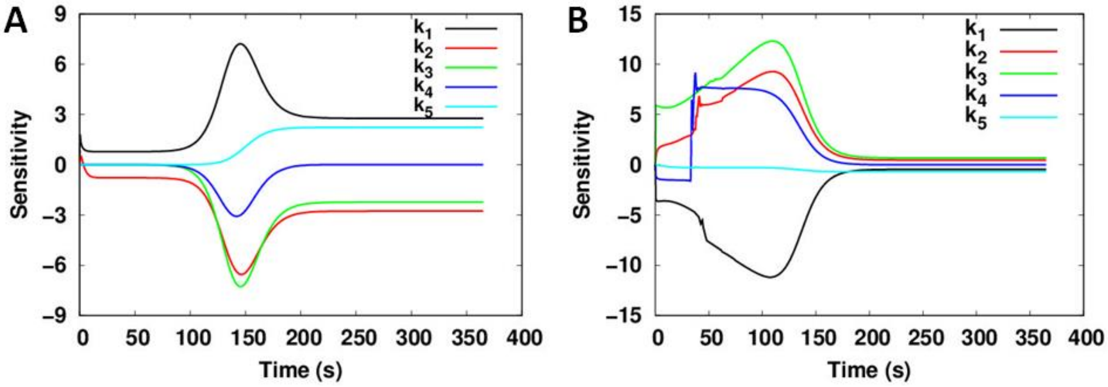

Figure S12. Sensitivity coefficients as a function of time for kinetic Model 2 for **(A)** the J-type nanostructure and **(B)** the H-type nanostructure. The $\Gamma_{[ZnTPPc]}$ value was 4.0 nmol·cm^{-2}.

PCA was applied in order to determine the number of significant components in the dataset presented in Figure 6B (main text) where the aqueous citric acid concentration is set at 50 mM. From the scree plot in Figure S13A, the eigenvalues associated to the first component explain more than 97% of data variance. This result is further confirmed by analyzing the scores and loadings from the PCA (Figure S13B-C). Although, from a simple inspection of Figure 6B the presence of more than one species is clearly discerned, the overlapping is so severe that a curve resolution will not successfully resolve the data. Based on these considerations, no MCR-ALS was performed on the datasets in Figure 6 involving large concentrations (50, 100 and 250 mM) of aqueous citric acid electrolyte.

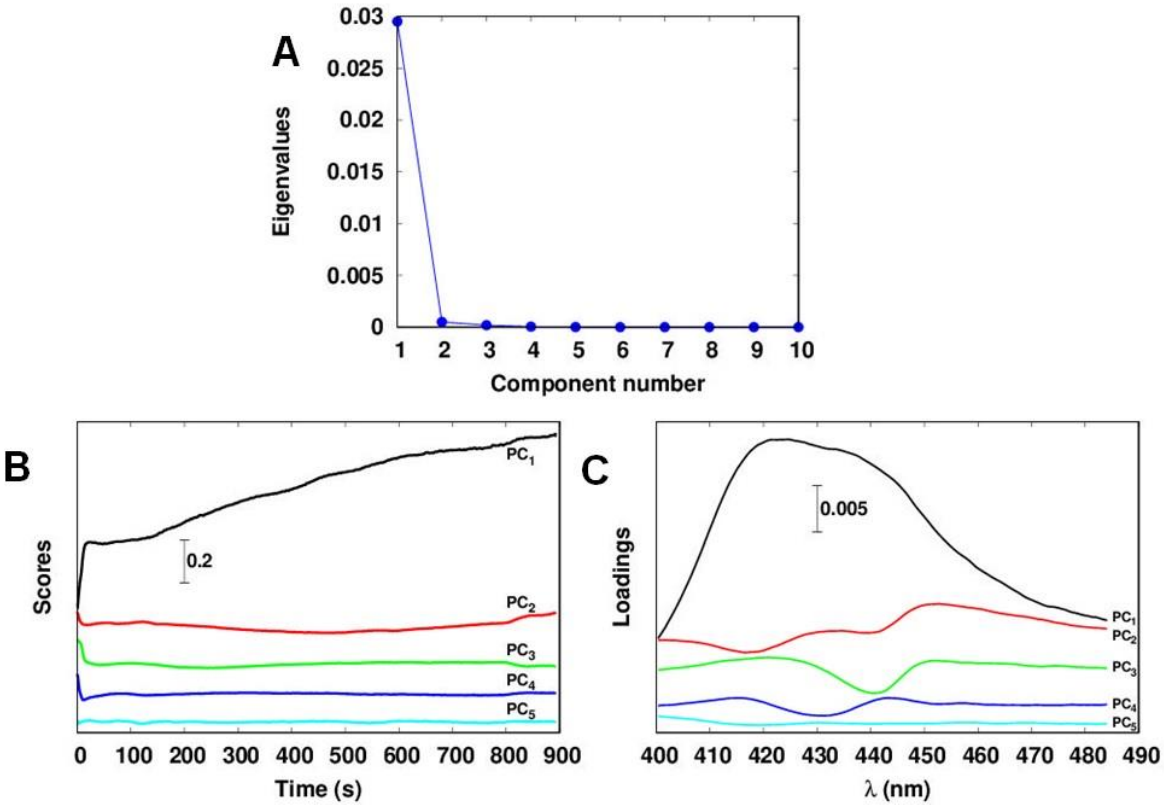

Figure S13. Scree plot and representation of the PCA results for the TIR-UV/vis dataset reported in Figure 6B (main text). **(A)** Scree plot, **(B)** PCA scores as function of time and **(C)** PCA loadings as a function of wavelength.

The microscopic morphology of the interfacial nanostructures using 10 and 100 mM aqueous citric acid electrolyte was probed using AFM (Figures 7C-D and 7G-H, main text). AFM survey scans in semi-contact mode of 10 µm x 10 µm were initially performed to investigate the surface roughness and optimize the scan parameters. The measured RMS and R_a roughness values of the H-type nanostructures (when using 10 mM citric acid) were 32.10 and 23.61 respectively. The measured RMS and R_a roughness values of the J-type nanostructures (when using 100 mM citric acid) were 5.97 and 4.35 respectively.

Lower resolution scans of 0.5 µm x 0.5 µm and 1.0 x 1.0 µm were performed on areas of interest in Figures 7D and 7H, respectively. Here, for the H-type nanostructures, the RMS and R_a values were 9.12 and 5.38. The high roughness is due to the scan taking place over the edge of a flake. The J-type nanostructures had RMS and R_a values of 0.73 and 0.59. The reduced roughness of the J-type nanostructures is due to the apparent absence of flakes. This information is summarized in Table S7. These results agree with the images obtained by SEM (Figures 7A-B and 7E-F, main text).

Table S7. Summary of AFM parameters

**Sample**	**H-type nanostructures**		**J-type nanostructures**	
**Area (µm x µm)**	5.0 x 3.5	0.5 x 0.5	5.0 x 5.0	1.0 x 1.0
**RMS**	32.01	9.12	5.97	0.73
**R_a**	23.61	5.38	4.35	0.59

S5. SUPPLEMENTARY REFERENCES

(1) Romeo, A.; Castriciano, M. A.; Occhiuto, I.; Zagami, R.; Pasternack, R. F.; Scolaro, L. M. Kinetic Control of Chirality in Porphyrin J-Aggregates. *J. Am. Chem. Soc.* **2014**, *136* (1), 40–43. https://doi.org/10.1021/ja410514k.

(2) Molina-Osorio, A. F.; Cheung, D.; O'Dwyer, C.; Stewart, A. A.; Dossot, M.; Herzog, G.; Scanlon, M. D. Self-Assembly of Porphyrin Nanostructures at the Interface between Two Immiscible Liquids. *J. Phys. Chem. C* **2020**, *124* (12), 6929–6937. https://doi.org/10.1021/acs.jpcc.0c00437.

(3) R Core Team (2013). R: A language and environment for statistical computing. R Foundation for Statistical Computing, Vienna, Austria. URL https://www.R-project.org/.

(4) Liland, K. H.; Almøy, T.; Mevik, B. H. Optimal Choice of Baseline Correction for Multivariate Calibration of Spectra. *Appl. Spectrosc.* **2010**, *64* (9), 1007–1016. https://doi.org/10.1366/000370210792434350.

(5) Tauler, R.; Smilde, A.; Kowalski, B. Selectivity, Local Rank, Three-way Data Analysis and Ambiguity in Multivariate Curve Resolution. *J. Chemom.* **1995**, *9* (1), 31–58. https://doi.org/10.1002/cem.1180090105.

(6) De Juan, A.; Jaumot, J.; Tauler, R. Multivariate Curve Resolution (MCR). Solving the Mixture Analysis Problem. *Anal. Methods* **2014**, *6* (14), 4964–4976. https://doi.org/10.1039/c4ay00571f.

(7) Ruckebusch, C.; Blanchet, L. Multivariate Curve Resolution: A Review of Advanced and Tailored Applications and Challenges. *Anal. Chim. Acta* **2013**, *765*, 28–36. https://doi.org/10.1016/j.aca.2012.12.028.

(8) Lê, S.; Josse, J.; Husson, F. FactoMineR : An R Package for Multivariate Analysis. *J. Stat. Softw.* **2008**, *25* (1), 253–258. https://doi.org/10.18637/jss.v025.i01.

(9) Mullen, K.M. (2015). ALS: Multivariate Curve Resolution Alternating Least Squares (MCR-ALS). R package version 0.0.6. https://CRAN.R-project.org/package=ALS.

(10) Windig, W.; Stephenson, D. A. Self-Modeling Mixture Analysis of Second-Derivative Near-Infrared Spectral Data Using the Simplisma Approach. *Anal. Chem.* **1992**, *64* (22), 2735–2742. https://doi.org/10.1021/ac00046a015.

(11) Frieden, C.; Goddette, D. W. Polymerization of Actin and Actin-like Systems: Evaluation of the Time Course of Polymerization in Relation to the Mechanism. *Biochemistry* **1983**, *22* (25), 5836–5843. https://doi.org/10.1021/bi00294a023.

(12) Soetaert, K.; Petzoldt, T. Inverse Modelling, Sensitivity and Monte Carlo Analysis in R Using Package FME. *J. Stat. Softw.* **2010**, *33* (3), 1–28. https://doi.org/10.18637/jss.v033.i03.

(13) Vosough, M.; Mason, C.; Tauler, R.; Jalali-Heravi, M.; Maeder, M. On Rotational Ambiguity in Model-Free Analyses of Multivariate Data. *J. Chemom.* **2006**, *20* (6–7), 302–310. https://doi.org/10.1002/cem.1022.

(14) Abdollahi, H.; Maeder, M.; Tauler, R. Calculation and Meaning of Feasible Band Boundaries in Multivariate Curve Resolution of a Two-Component System. *Anal. Chem.* **2009**, *81* (6), 2115–2122. https://doi.org/10.1021/ac8022197.

(15) Haario, H.; Laine, M.; Mira, A.; Saksman, E. DRAM: Efficient Adaptive MCMC. *Stat. Comput.* **2006**, *16* (4), 339–354. https://doi.org/10.1007/s11222-006-9438-0.

(16) Molina-Osorio, A. F.; Manzanares, J. A.; Gamero-Quijano, A.; Scanlon, M. D. Electrochemically Controlled Ion Dynamics in Porphyrin Nanostructures. *J. Phys. Chem. C* **2020**, *124* (33), 18346–18355. https://doi.org/10.1021/acs.jpcc.0c04976.
